# Estimating the capacity for production of formamide by radioactive minerals on the prebiotic Earth

**DOI:** 10.1038/s41598-017-18483-8

**Published:** 2018-01-10

**Authors:** Zachary R. Adam, Yayoi Hongo, H. James Cleaves, Ruiqin Yi, Albert C. Fahrenbach, Isao Yoda, Masashi Aono

**Affiliations:** 1 Department of Earth and Planetary Sciences, Harvard University, Cambridge, MA USA; 2 Blue Marble Space Institute of Science, Seattle, WA USA; 3 Earth-Life Science Institute, Tokyo Institute of Technology, Tokyo, Japan; 4 Institute for Advanced Study, Princeton, NJ 08540 USA; 5 Center for Chemical Evolution, Georgia Institute of Technology, Atlanta, GA 30332 USA; 6 Faculty of Environment and Information Studies, Keio University, Kanagawa, Japan

## Abstract

Water creates special problems for prebiotic chemistry, as it is thermodynamically favorable for amide and phosphodiester bonds to hydrolyze. The availability of alternative solvents with more favorable properties for the formation of prebiotic molecules on the early Earth may have helped bypass this so-called “water paradox”. Formamide (FA) is one such solvent, and can serve as a nucleobase precursor, but it is difficult to envision how FA could have been generated in large quantities or accumulated in terrestrial surface environments. We report here the conversion of aqueous acetonitrile (ACN) via hydrogen cyanide (HCN) as an intermediate into FA by γ-irradiation under conditions mimicking exposure to radioactive minerals. We estimate that a radioactive placer deposit could produce 0.1‒0.8 mol FA km^−2^ year^−1^. A uraninite fission zone comparable to the Oklo reactors in Gabon can produce 0.1‒1 mol m^−2^ year^−1^, orders of magnitude greater than other scenarios of FA production or delivery for which reaching sizeable concentrations of FA are problematic. Radioactive mineral deposits may be favorable settings for prebiotic compound formation through emergent geologic processes and FA-mediated organic chemistry.

## Introduction

Abiotically produced nucleic and amino acid polymers are widely viewed as key chemical intermediates that link a lifeless Earth with the universal ancestor of all known life^1^. Laboratory studies, however, have revealed several problems with the abiotic synthesis of these polymers prior to life’s emergence. Specifically, though the prebiotic synthesis of RNA monomers has recently been reported from relatively simple molecules^2–5^, only a few geochemical scenarios that can facilitate these synthesis reactions have been experimentally investigated^6,7^. A frequent criticism of many model prebiotic reactions is that it is unlikely the needed precursor ingredients would ever spontaneously reach the high concentrations typically employed, giving rise to a puzzle known as the Concentration Problem^8^. But perhaps the most deleterious barrier to the emergence of life arises from the most critical ingredient of life itself ‒ water^9^. The average half-life for hydrolysis of a peptide bond is on the order of 10^2^ years at pH 7 and 25 °C^10^, and the half-life of an RNA phosphodiester bond at 30 °C is estimated to be less than one year under the same conditions^11^. Water therefore hydrolyzes biopolymers such as nucleic acids and proteins over relatively short time-scales, but is also a necessary solvent medium for all known life. This fundamental mismatch between initial and final chemical constraints on life’s origins leads to what is known as the Water Paradox^12^. The Concentration Problem and the Water Paradox present formidable conceptual problems for any origins of life theory, including “RNA World” scenarios that necessitate abundant, abiotic production of polynucleotides^2^.

Formamide (HCONH_2_, FA), which is a liquid under normal terrestrial surface temperature and pressure conditions, has been advanced as an alternative solvent to water that could enable chemical complexification and a means of bypassing the Water Paradox^13–15^. FA is a polar solvent that has the advantageous properties of promoting dehydration condensation reactions^16,17^, solubilizing phosphate minerals^18^, and serving as a feedstock for several biologically relevant compounds including nucleobases^19–21^, amino acids and carboxylic acids when heated and in contact with a variety of mineral catalysts^5,22–25^. FA can be produced via the hydration of hydrogen cyanide (HCN) or the decomposition of ammonium formate, but the competing reaction of FA hydrolysis to formic acid constrains maximum possible aqueous concentrations to approximately 10^−5^ M^26–28^. These dilute amounts are insufficient to validate the use of neat concentrations of FA employed in some model prebiotic syntheses, or to serve as a reservoir for abundant nucleobase production^29–32^. Other direct sources of FA that bypass bodies of water such as cometary^33^ or meteoritic^34^ delivery have been invoked in conjunction with ‘desert-like’ periodically dry settings or hydrothermally-driven thermophoresis as a means of concentrating FA^9,27,29,30,35^, but such scenarios necessitate ideal and perhaps implausible circumstances to simultaneously produce and then concentrate FA to any appreciable reservoir size^36^.

**Figure 1:**
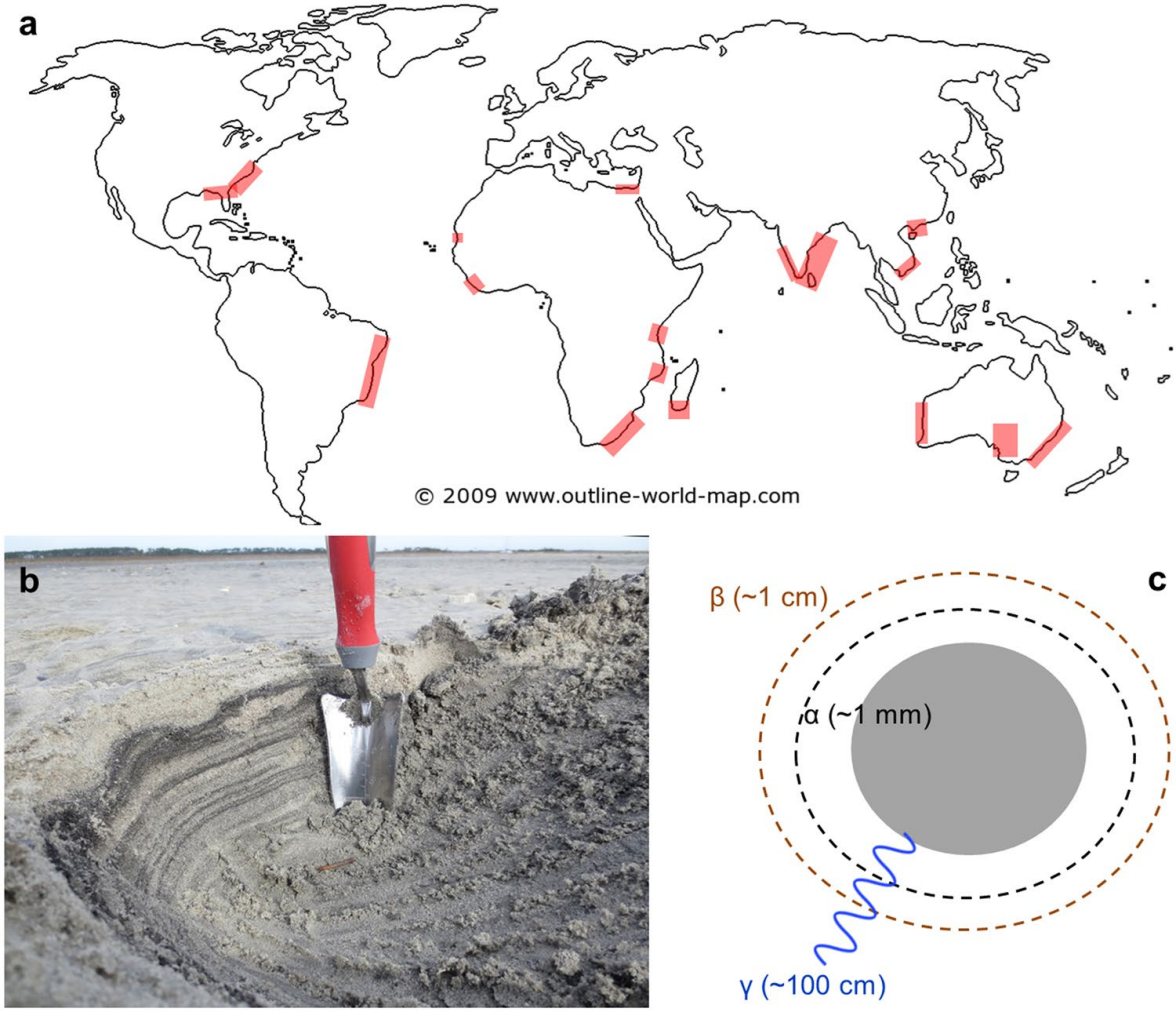
Factors causing heterogeneous distribution of energy from radioactive placer minerals. (**a**) Global distribution of major modern heavy mineral placer deposits. (**b**) Concentration of heavy mineral grains (dark layers) within a typical beach placer deposit; image courtesy C. Bern, United States Geological Survey. (**c**) Spatial distribution of α, β and γ particles around a single radioactive mineral grain.

A localized geochemical source of FA via radiolysis of aqueous nitriles such as HCN or acetonitrile (ACN) may reduce problems associated with reaching high concentrations of FA while simultaneously mitigating the Water Paradox. Radiolysis of water produces hydroxyl radicals that increase rates of hydration and hydrolysis above those associated with thermal activation alone^37^. Radioactive minerals such as monazite, uraninite, and zircon are concentrated by hydrodynamic sorting in beach and river settings called placer deposits. Placers are widely distributed but heterogeneously concentrated across the Earth’s surface^38–40^ (Fig. 1a,b). The energy from radioactive minerals create fluxes of α, β and γ particles that are orders of magnitude above background levels near the surfaces of individual mineral grains (Fig. 1c). Under exceptional circumstances, uranium-rich strata formed in the Earth’s deep past created self-sustaining neutron chain reactions with radiolytic and heat power outputs that greatly exceed those of typical placer deposits^41–44^, a unique energy setting with prebiotic implications explored in detail by Draganic *et al*.^45,46^ and most recently expanded upon by Maruyama and Ebisuzaki^47^. Here, we investigate the hypothesis that γ radiolysis of atmospherically-derived aqueous nitriles by radioactive minerals could have increased production rates of FA and generated sizeable organic solvent reservoirs on the prebiotic Earth.

## Results

**Figure 2:**
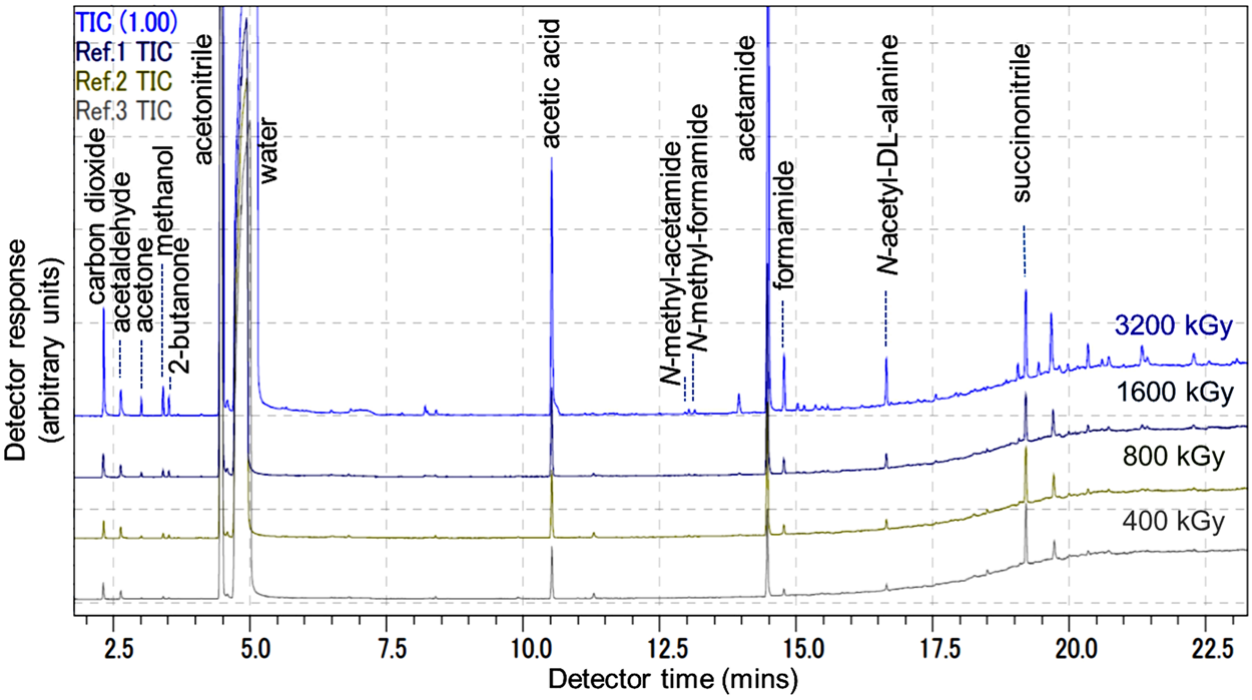
GC-MS chromatogram of the most abundant identified products (annotated peaks) resulting from gamma irradiation of mixtures of 30% aqueous ACN. Indicated total doses were delivered at a dose rate of ~3 kGy hr^−1^.

**Figure 3:**
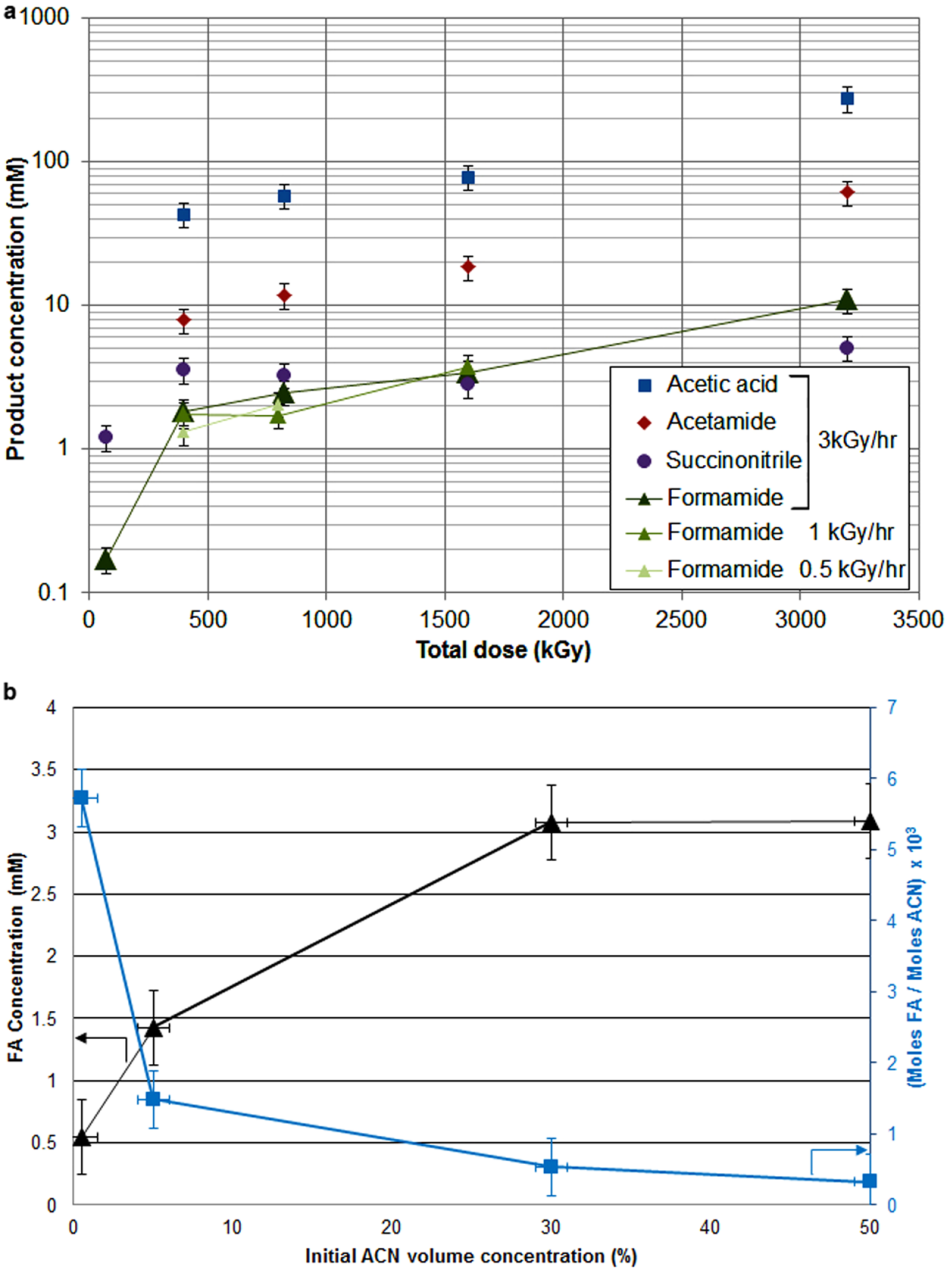
FA production as a function of total dose and yield from ACN. (**a**) Concentration of compounds at 2.92, 1.0 and 0.5 kgry hr^−1^ γ-dose rates versus total dose. (**b**) FA yield (triangles, mM) and molar conversion efficiency (squares, dimensionless) versus initial acetonitrile concentration for 800 kGy total dose at ~3kGy hr^−1^ dose rate.

The irradiation of ACN was initially investigated as a 30 volume % (~5.7 M) aqueous solution, which was exposed to an 800 kGy dose at 3kGy h^−1^. GC-MS was used to analyze the resulting mixture. FA was observed along with acetaldehyde, acetamide, acetic acid and succinonitrile as major products (Fig. 2). Hydrogen cyanide (HCN) was detected by GC-MS analysis of the headspace of the reaction vial. Using a calibration curve determined from known concentrations of an authentic standard, a concentration of 6.6 mM FA was measured (Fig. 3a, SI-1). The decrease in concentration of ACN due to conversion to other products was not detectable within experimental error, consistent with the relatively low yields of organic products detected. We then examined the effects of dose rate, total dose and initial concentration of ACN in order to constrain the production of FA under varying conditions. The measured concentrations of FA using 30% ACN aqueous solutions rapidly increase between 0 and 400 kGy, then increase in an approximately linear manner with total dose without significant variation as a function of dose rate (green lines, Fig. 3a). The same general trend was observed for acetic acid, acetamide and succinonitrile. From these data, we calculated a radiolytic yield (*G*) for FA formation from ACN of 0.012 molecules/100 eV. Although the radiolytic yield for FA decreases with lower starting concentrations of ACN, normalizing the *G*-values to initial ACN molarity reveals that the production of FA becomes more efficient at lower initial ACN concentrations (Fig. 3b). Overall, the conversion of aqueous ACN to FA is robust to varying dose rate (measured from 0.5‒2.92 kGy h^−1^), total dose (over the range of 72 to 3200 kGy) and initial concentration of ACN (0.5‒50 volume %). At 3200 kGy total dose, additional compounds including *N*-methyl-acetamide, *N*-methyl-formamide and pyrimidine-like compounds were produced at levels that could not be detected at lower total doses (Table SI-1). Generation of FA seems robust to oxidizing conditions; replacing N_2_ with a standard atmosphere or adding oxygen-rich nitrate had only a minor effect on FA production.

Given the observations described above, we infer a pathway of FA production that proceeds though HCN as an intermediate. The radiolytic generation of HCN from pure ACN has been previously reported, the mechanism of which is proposed to proceed by direct photolysis of ACN generating •CN and •CH_3_ radicals by homolytic cleavage^48^.

$${{\rm{CH}}}_{{\rm{3}}}{\rm{CN}}+hv\to \bullet {\rm{CN}}+\bullet {{\rm{CH}}}_{{\rm{3}}}$$

Subsequent abstraction of a proton from ACN, or in our case from a molecule of water, furnishes the HCN molecule.

Following the production of HCN, the generation of FA follows a well-understood mechanism^49^. Addition of a hydroxyl radical followed by rapid tautomeric rearrangement leads to the FA radical.

$$\bullet {\rm{OH}}+{\rm{HCN}}\to \bullet {\rm{NCHOH}}\to \bullet {\rm{OCHNH}}$$

Protonation and electronic disproportionation with another radical species leads to the FA molecule. The fact that addition of oxygen or nitrate, i.e., electron scavengers, had only a minor effect on the yield of FA also suggests that •OH radicals are involved in the mechanism. Radiolysis of aqueous 25 mM NaCN at pH 7 at 2.92 kGy h^−1^ over a range of dose rates revealed FA as a major product detectable by GC-MS (*G* = 0.08 molecules/100 eV), corroborating the proposed mechanism of FA formation through an HCN intermediate.

## Discussion

**Figure 4:**
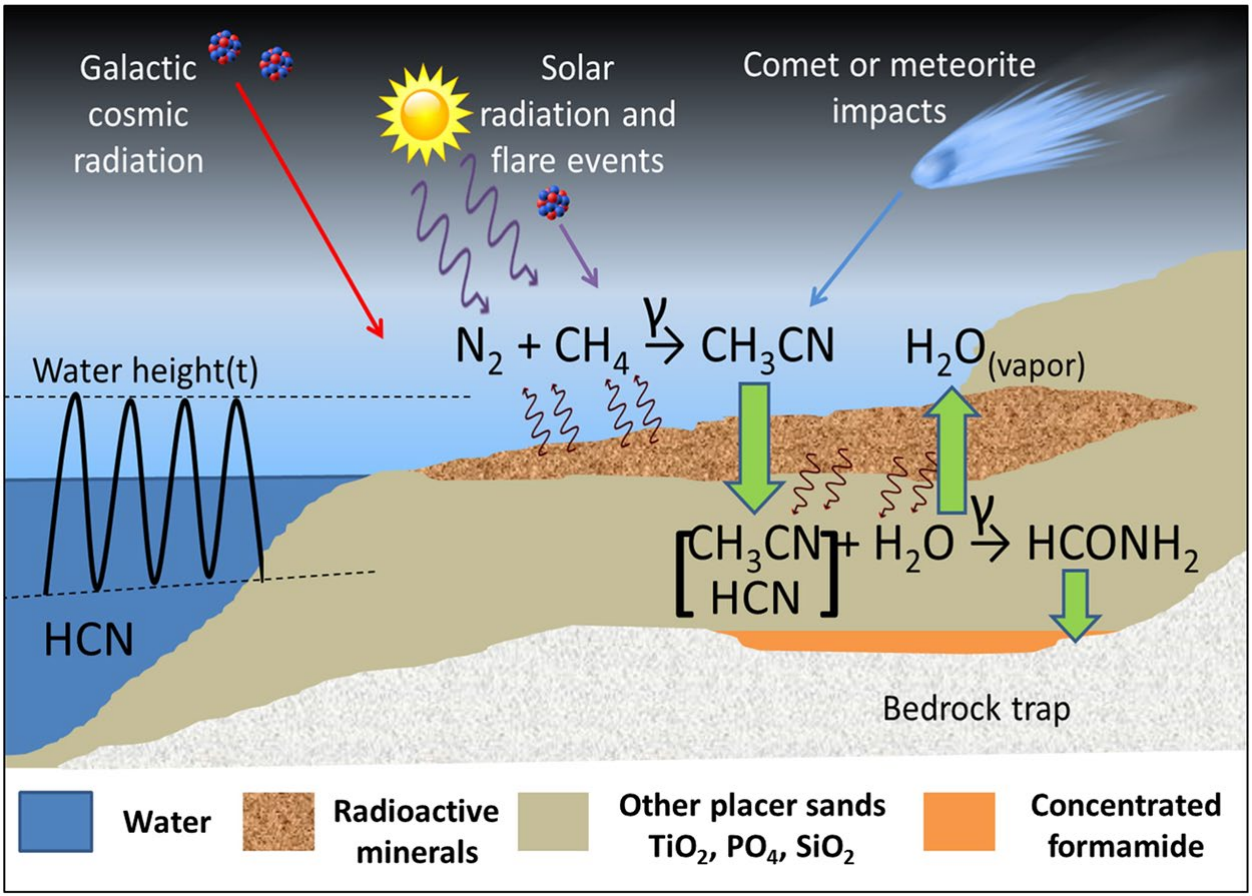
Proposed formamide synthesis and concentration process, depicting localized production and concentration of formamide (HCONH_2_) near radioactive mineral deposits on terrestrial surface environments. Note that chemical reactions are not balanced for simplicity.

Radiolysis of aqueous ACN and HCN produces amide and nitrile solvents at rates that exceed hydrolytic decomposition. These experiments simulate terrestrial synthesis pathways that produce FA without invoking extraterrestrial influx or thermally mediated hydrolysis of HCN. Experiments have shown that radiolysis of N_2_ and CH_4_ yield HCN and ACN as two major products^50,51^; though HCN appears to be the crucial intermediate to FA production, it is also a more volatile and readily reactive compound, which poses a challenge to having it accumulate to significant concentrations. ACN, on the other hand, is much less volatile, affording it the potential to settle from the atmosphere and to collect in surface water reservoirs along with other, less abundant nitrile species^52^. Nitriles produced by gamma radiolysis of atmospheric N_2_ and CH_4_ proximal to radioactive minerals could have been supplemented by more distributed energy sources such as solar UV, galactic cosmic rays (GCRs) or coronal mass ejections associated with solar flare events^53^ throughout the atmospheric column, which would have been introduced into the system during periodic influx of external water. The result would have been robust, long-term production of FA in a fixed setting (Fig. 4).

Repetitive wet/dry and heat/cooling cycles, occurring over a long duration of time, seem to be a prerequisite for FA-assisted prebiotic scenarios^27^. Heat dissipation associated with fissioning mineral seams could have assisted in the rapid concentration of FA. Laboratory FA-based prebiotic synthesis experiments employ concentrated FA or relatively high temperatures (>100 °C)^26,31,54,55^. Temperature fluctuations caused by water- or organic-moderated fission of uranium-bearing minerals would periodically raise temperatures above 100 °C, while also permitting influx of new sources of dissolved HCN or ACN from external sources that could be radiolyzed to produce FA. To determine if the concentration of FA is possible in the presence of the other radiolysis products observed (Fig. SI2)^30^, we carried out a simple concentration experiment by heating a 450 mL solution containing 250 mM each of FA, acetic acid, formic acid, acetamide and succinonitrile in an oven above a temperature of 100 °C. The concentrations of these molecules were evaluated as a function of time using ^13^C NMR spectroscopy (Fig. SI3). After approximately one day, all of the water had evaporated leaving behind a desiccated mixture in approximately the same molar proportions of starting materials.

The radiolysis yields determined herein enable estimation of the geochemical production parameters of FA within a radioactive mineral deposit. The *G* values describing the production of FA from ambient ACN and HCN are derived from this study. A typical sandstone sediment has pores that make up about 20% of the total volume^56^, wherein radiolytic chemistry may occur; calculated productivity is therefore reduced by approximately this proportion. The radioactivity associated with heavy mineral placer sands^38,39^ yields about 0.1–0.8 mol FA km^−2^ yr^−1^. This is over an order of magnitude greater than area- and time-averaged delivery from extraterrestrial sources^33,34^, with the added advantage that FA is produced within a relatively small volume proximal to the radioactive deposit (thus reducing reliance upon an external concentration mechanism). A fissioning mineral seam of uranium, with dimensions comparable to a typical fission zone described from the Paleoproterozoic Oklo deposit, has a combined (radiolysis and heat) power output of about 10 kilowatts^42,46,57,58^. About 13% of this power is composed of γ or β rays that can penetrate substantial distances beyond their host minerals^59^. These parameters yield an estimated FA production rate of 0.1–1 moles FA m^−2^ yr^−1^, which is over 6 orders of magnitude larger than the estimated FA production from placer minerals or delivery from cometary material per unit area^34^. The highest area-normalized production rate of about 1 mole FA m^−2^ yr^−1^ would require conversion of approximately 10% per day of the HCN present within the penetration range of the mineral seam’s γ radiation, and with HCN at the highest estimated concentration of oceanic reservoirs; a smaller conversion fraction per day would be required for shallow lake reservoirs with higher HCN concentrations^26^. This estimate does not include direct HCN or ACN production from irradiation of atmospheric gases by the deposit itself, nor does it include diffusion of HCN or ACN from adjacent volumes each day. Within this envelope of parameters, the theoretical upper limit of FA production in shallow terrestrial settings would be limited by efficiency of radiolytic conversion from HCN and ACN feedstocks rather than by the amount of nitrile feedstock abundance. An individual fission zone comparable to those at Oklo could produce an upper limit of 230,000 kg of FA over an average fission zone lifespan of about 500,000 years and within a radius of about 10 meters.

**Figure 5:**
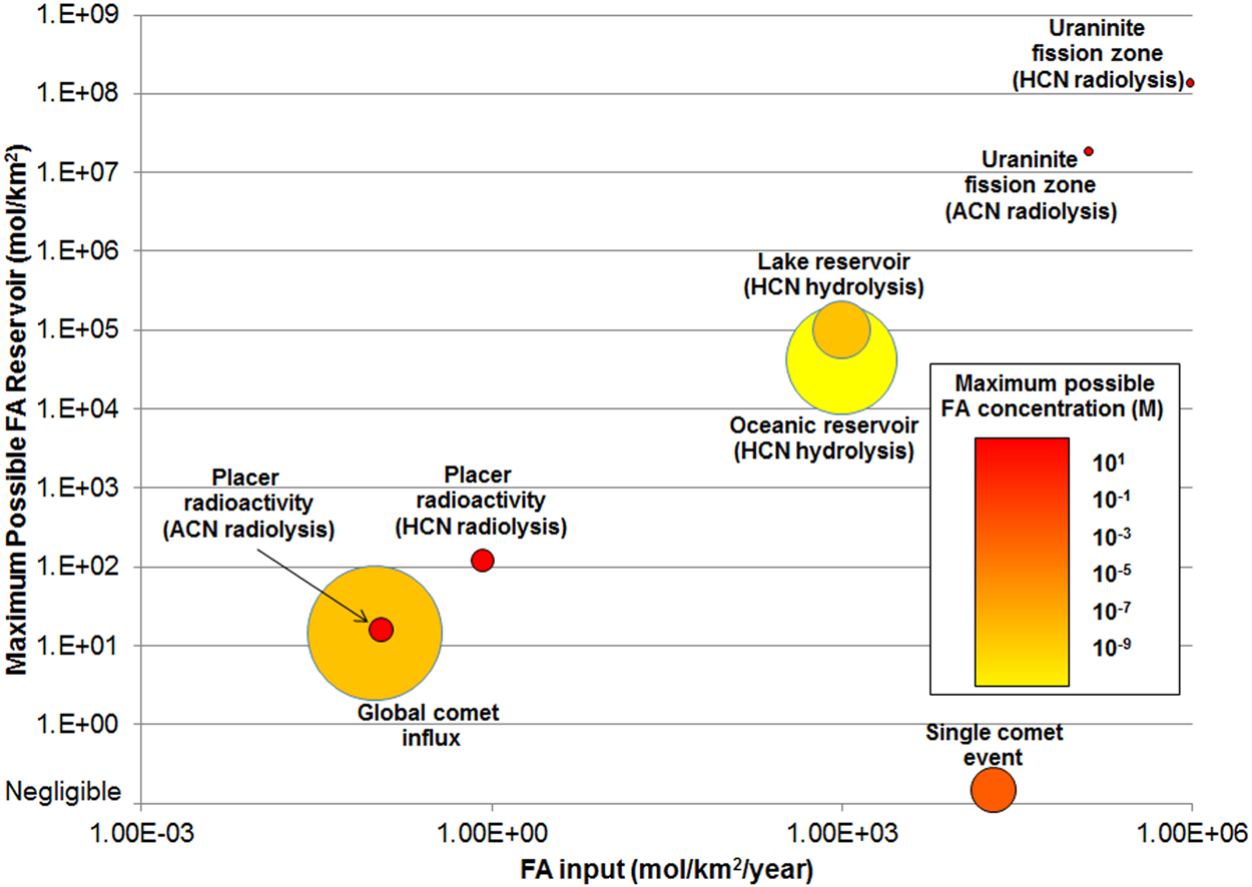
Formamide (FA) maximum reservoir size for different FA delivery or production mechanisms. FA sources include global comet influx^34^, a single comet event^33^, ideal pH and temperature for oceanic and lake reservoirs from HCN hydrolysis^26^, and radiolysis of ACN and HCN within radioactive placers and uraninite fission zones. Marker size indicates characteristic area of reservoir, with smaller markers indicating more localized processes. Heat map indicates maximum possible FA concentration of the reservoir.

The maximum possible area-normalized reservoir capacities for each mechanism of FA input can be estimated based on production figures and energy input associated with these settings (Fig. 5). If prevailing surface conditions are assumed to induce an FA degradation rate of about 0.7% per year on all potential reservoirs (*i.e*., FA degradation in water at 30 °C and pH 7^26^) then each maximum area-normalized reservoir is calculated as the ratio of normalized FA input to the degradation rate (Fig. SI4). Isolated comet impacts have a maximum reservoir size of approximately zero, because input is a step function rather than a continuous flux, and therefore FA degradation after the impact event cannot be counterbalanced except through some combination of the other input processes. Steady-state production and degradation of FA in oceanic and shallow lake reservoirs are not capable of reaching neat FA concentrations required to undergo reactions of prebiotic interest^26^. Among all considered FA sources that have been reported, the radiolysis of ACN and HCN within uranium fission zones have the highest area-normalized FA production rate and reservoir size, and the smallest characteristic source areas.

Radioactive mineral deposits capable of producing abundant FA would place this compound in contact with placer minerals relevant to prebiotic chemistry. Common placer sediments include monazite ((Sm, Gd, Ce, Th)PO_4_), rutile (TiO_2_), pyrite (FeS_2_) and apatite (Ca_10_(PO_4_)_6_(OH)_2_)^40^, some of which promote condensation of FA into nucleobases^14^. We observed herein that radiolysis of aqueous ACN produces significant amounts of four different amide solvents (FA, acetamide, *N*-methyl-acetamide and *N*-methyl-formamide), all of which are known to support the phosphorylation of nucleosides and nucleotides with solid phase apatite^60^, yielding multiple pathways and abundant sources of two of three nucleic acid monomer components: nucleobases and phosphate.

Radioactive mineral deposits are unique geochemical environments that can produce FA far more quickly than it is degraded. FA is a demonstrated nucleobase precursor and water-alternative solvent with favorable properties for phosphorylation and polymerization. In conjunction with localized production of abundant FA, these geochemical settings may offer one possible means of mitigating both the Water Paradox and the Concentration Problem, at least with respect to the production of nucleotide components. Periodic drying of placer deposits has the potential to concentrate FA, other high boiling-point solvents, and solutes to a degree which renders condensation reactions favorable. Radioactive mineral deposits open the intriguing possibility that water-alternative solvents could have assisted or even enabled the emergence of polymeric chemical systems that preceded water-based life as we know it.

## Methods

Reagents (including ACN, NaCN, FA, formic acid, acetamide and succinonitrile) were purchased from Sigma-Aldrich, Japan and were of 99% purity or higher and used without further purification. Purified 18 MΩ water produced by EMD Millipore’s Milli-Q® water purification system was loaded into glass vials which were pre-ashed at 500 °C for three hours to remove organic contaminants. In the case of ACN, the vials were frozen, vacuum purged and back filled with N_2_ three times. For HCN experiments, the solutions were prepared from NaCN and water which had been degassed with N_2_. The pH of the NaCN solutions were adjusted to 7 using concentrated HCl, after which the reaction vials were sealed and the solutions were subjected to further purging with N_2_ for a period of about five minutes to remove any residual O_2_. For each experiment, two sets of vials were created. One set was exposed to varying doses and dose rates of γ-radiation from a ^60^Co source (10‒30 cm distances, with dose rates varying from approximately 3.0‒0.5 kGry hr^−1^, respectively) at the Tokyo Institute of Technology’s ^60^Co Gamma Radiation Facility. These dose rates are roughly 75 times greater than that estimated from natural fission cores^46^. Another set of vials, which served as controls were kept in a darkened box at room temperature.

Volatiles in radiolysis samples were analyzed with a Shimadzu GCMS-QP2010 Ultra gas chromatrograph-mass spectrometer (GC-MS). Direct injection of liquid samples and head-space sampling were carried out using a PAL (prep and load solution) RTC autosampler. Data processing was conducted using the LabSolution_GCMSsolution version 4.11 software package. The injection method used an inlet temperature of 250 °C and a source temperature of 200 °C. The scan range was set to *m/z* 20–500 Da. Chromatography was conducted using an Rtx-WAX® column (60 m, 0.3 mm I.D., 0.5 mm df); helium was used as the carrier gas at a flow rate of 40 cm sec^−1^. The oven temperature program was: 2 min hold at 70 °C, from 70 to 240 °C at 10 °C min^−1^ and then the temperature was maintained at 240 °C for 5 min. Compounds were identified by comparison of their electron impact fragmentation spectra with standards from the NIST standard reference library, and by verification of spectra with off-the-shelf standard reagents. Concentrations were determined by establishing a calibration curve determined from known concentrations of authentic standards. FA heating and concentration experiment mixtures were analyzed in 10% D_2_O/90% H_2_O using a Bruker Avance III nuclear magnetic resonance (NMR) spectrometer tuned to measure ^13^C spectra at 100 MHz and 303 K.

### Data Availability

The datasets generated during the current study are available from the corresponding author on reasonable request.

## Electronic supplementary material

Supplementary Information
