# Supplementary material for: Estimating the capacity for production of formamide by radioactive minerals on the prebiotic Earth
Source: Sci Rep. 2018 Jan 10;8:265. doi: 10.1038/s41598-017-18483-8 (PMC5762809; doi:10.1038/s41598-017-18483-8)

SUPPLEMENTARY INFORMATION

Estimating the capacity for production of formamide by radioactive minerals on the prebiotic Earth

Zachary R. Adam[a,b,*], Yayoi Hongo[c], H. James Cleaves II[b,c,d,e], Yi Ruiqin[c], Albert C. Fahrenbach[c], Isao Yoda[c], and Masashi Aono[c,f]

[a] Department of Earth and Planetary Sciences, Harvard University, Cambridge, MA
[b] Blue Marble Space Institute of Science, Seattle, WA
[c] Earth-Life Science Institute, Tokyo Institute of Technology, Tokyo, Japan
[d] Institute for Advanced Study, Princeton, NJ 08540
[e] Center for Chemical Evolution, Georgia Institute of Technology, Atlanta, GA 30332
[f] Faculty of Environment and Information Studies, Keio University, Kanagawa, Japan

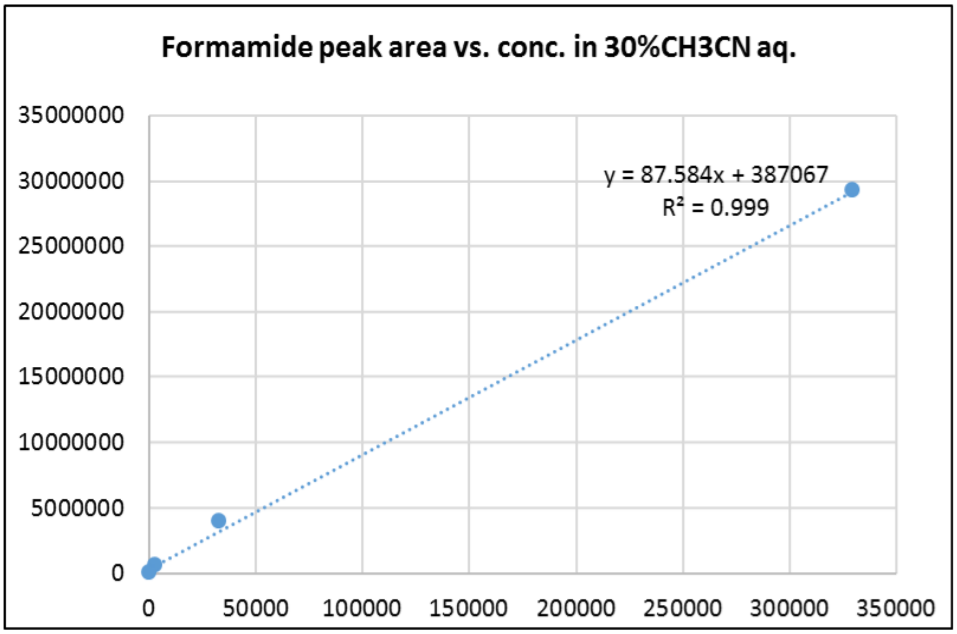

Formamide			
µM	area	height	
3E+05	3E+07	8E+06	
33000	4E+06	5E+05	
3300	6E+05		83336
660	peak ND		0
Detection limit according to the intercept		4419	µM
	area	conc µM	
sample LI No.4	9E+05	6322	
sample LI No.5	39381	<DL	
sample LI No.6	57504	<DL	
sample LI No.7	78299	<DL	
sample LI No.12	peak ND	ND	

Figure SI-1. Calibration curve for FA concentration determination.

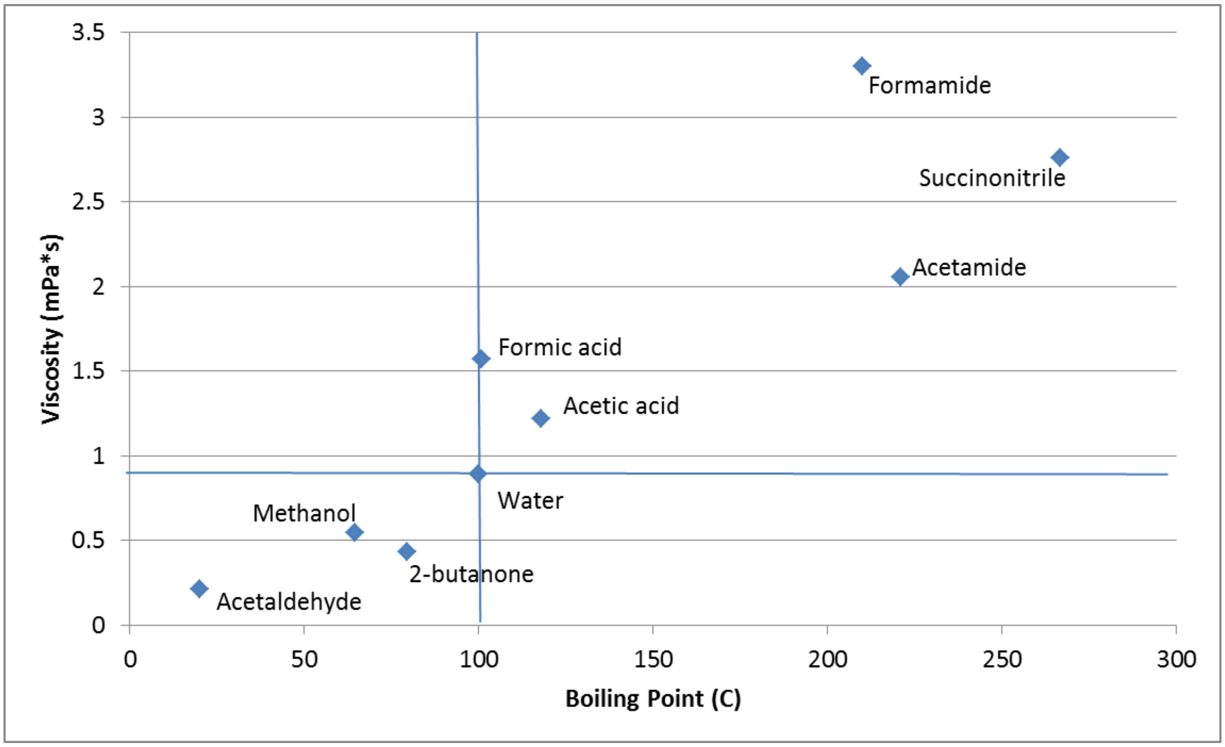

Figure SI-2. Boiling point and viscosity of aqueous ACN radiolysis products. Compounds in the upper right quadrant are more likely to remain when water is driven from the system.

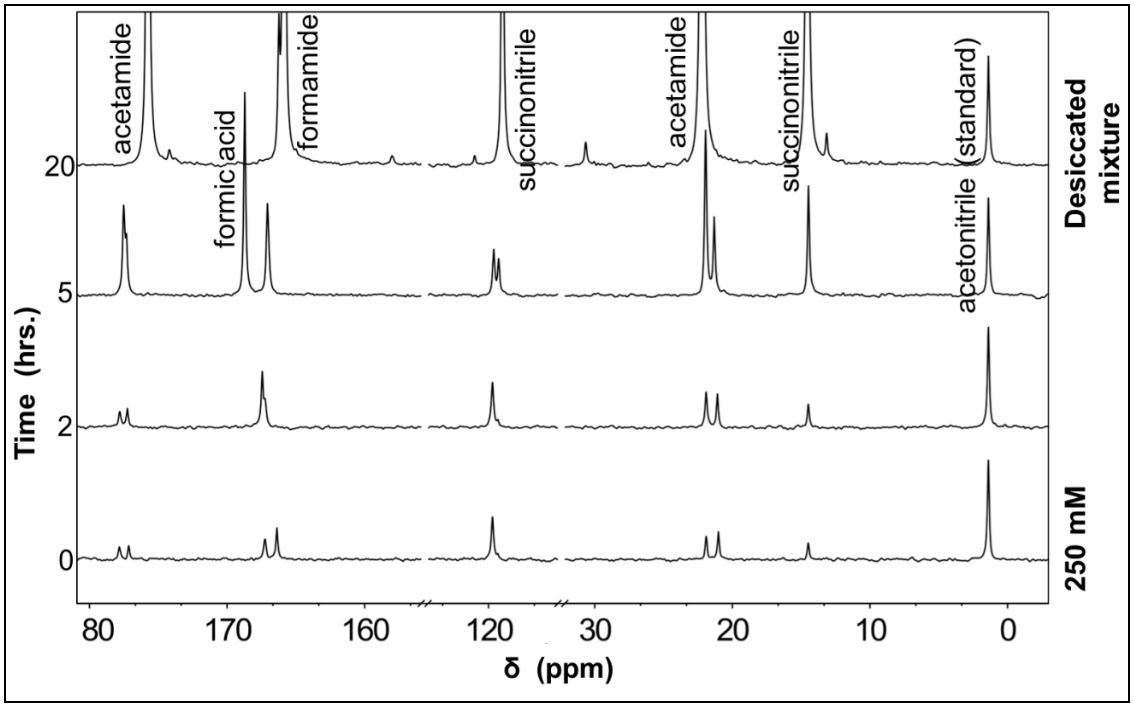

Figure SI-3. ^{13}**C-NMR results demonstrating concentration by evaporation of water from an aqueous mixture of major ACN radiolysis products heated at >100°C over 20 hours.**

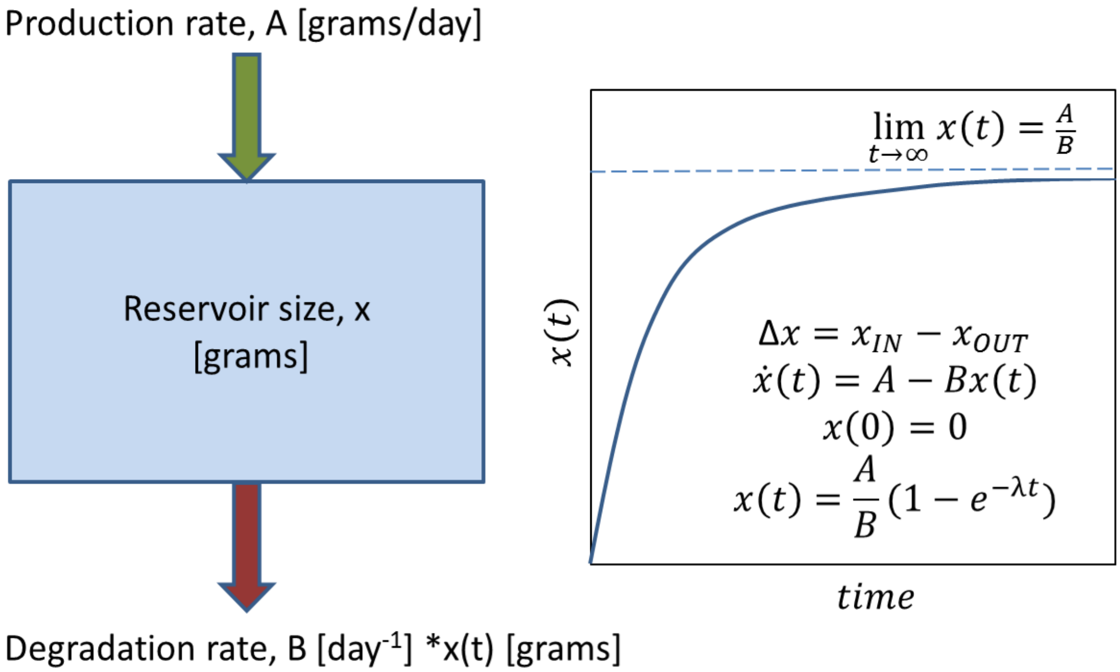

Figure SI-4. A simple box model of FA production incorporating a constant production rate A (in units FA per unit time) and a proportional degradation rate B (in percent degradation per unit time) limits the maximum FA reservoir size to the ratio A/B. FA production rates associated with atmospheric synthesis [20] and cometary influx [28] are simply too low to allow the buildup of FA because degradation rates are too high under plausible ambient conditions.

Table SI-1. Radiolysis product list for 30% acetonitrile and water at 2.92kGy/hr and 3200kGy total dose.

Compound number	Retention time (min)	Compound	Similarity Index
1	2.315	carbon dioxide	95
2	2.635	acetaldehyde	96
3	3.01	acetone	92
4	3.41	methyl alcohol	96
5	3.51	2-butanone	96
6	3.68	ethanol	88
7	4.105	2,3-butandedione	90
8	4.425	acetonitrile	94
9	5.66	butanenitrile	87
10	6.485	2-butenenitrile	89
11	7.775	acetaldoxime	87
12	8.205	1,3,5-triazine,2,4,6-trimethyl-	95
13	8.405	acetoin	87
14	10.565	acetic acid	92
15	13.045	acetamide,N-methyl-	91
16	13.145	formamide,N-methyl-	92
17	14.47	acetamide	96
18	14.78	formamide	94
19	15.04	propanamide	92
20	15.145	2-methyl-3-oxobutyronitrile	86
21	15.355	5-oxohexanenitrile	90
22	15.58	2-hexanol	85
23	16.655	DL-alanine,N-acetyl	89
24	16.74	propanenitrile,3-hydroxy-	89
25	19.065	1H-imidazole,1,2-dimethyl	92
26	19.205	succinonitrile	97
27	19.44	6-methyluracil	81
28	19.675	3,4,5-trimethylpyrazole	90
29	20.345	propanamide,2-hydroxy	92
30	21.335	uracil analog?	79
31	22.285	carbamic acid, methyl ester	86

Fragmentation spectra for compounds in Table SI-1. Note highlighted locations of retention time, compound name and similarity index in first two compound fragmentation spectra images.

Compound 1

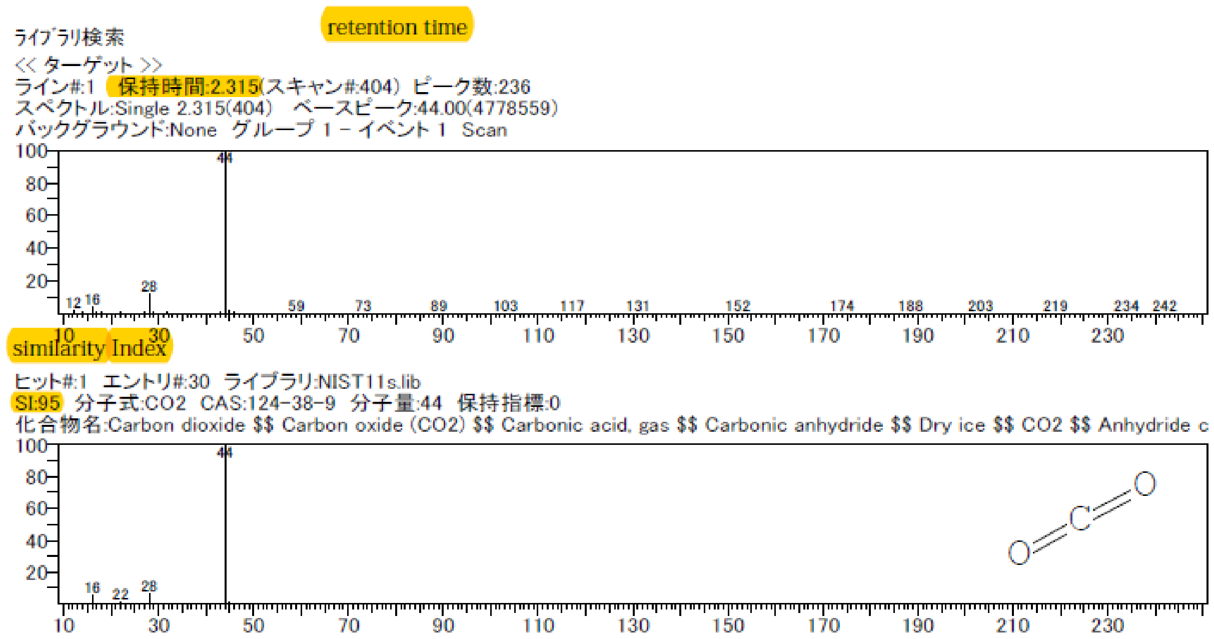

Compound 2

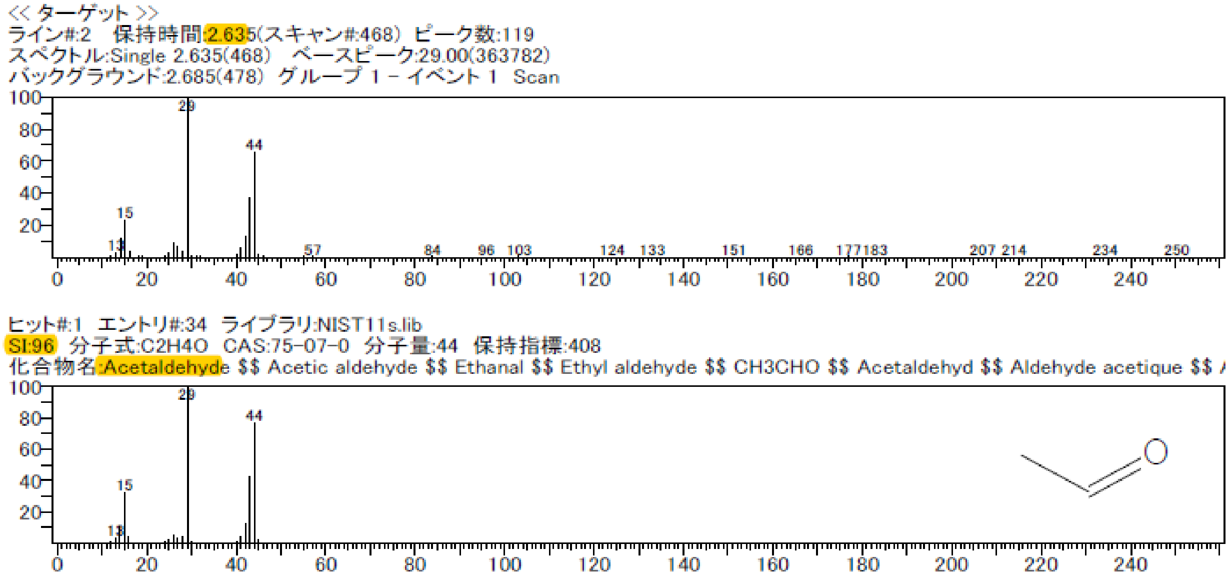

Compound 3

<< ターゲット >>
ライン#:3　保持時間:3.010(スキャン#:543)　ピーク数:239
スペクトル:Single 3.010(543)　ベースピーク:43.00(427330)
バックグラウンド:None　グループ 1 - イベント 1　Scan

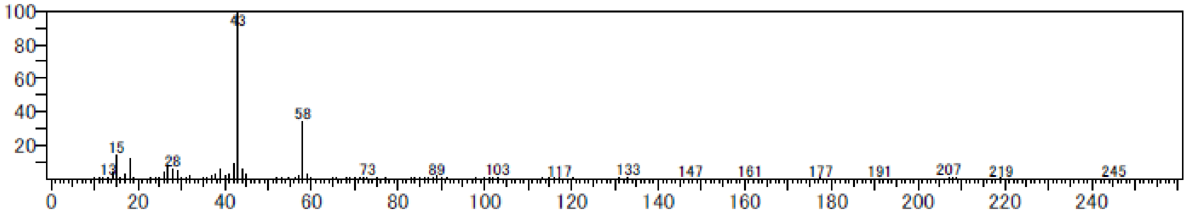

ヒット#:1　エントリ#:113　ライブラリ:NIST11s.lib
SI:92　分子式:C3H6O　CAS:67-64-1　分子量:58　保持指標:455
化合物名:Acetone $$ 2-Propanone $$ .beta.-Ketopropane $$ Dimethyl ketone $$ Dimethylformaldehyde $$ Methyl ketone $$ Pr

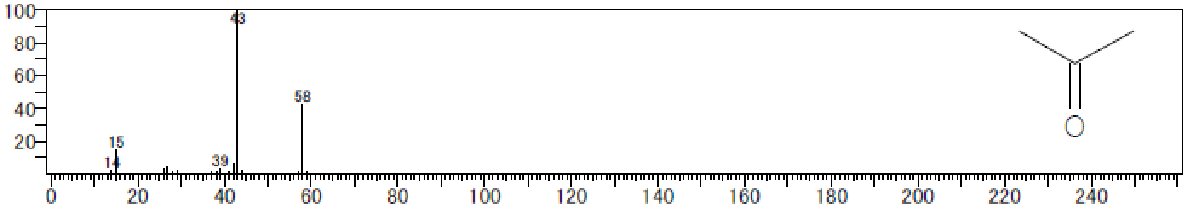

Compound 4

<< ターゲット >>
ライン#:4　保持時間:3.410(スキャン#:623)　ピーク数:128
スペクトル:Single 3.410(623)　ベースピーク:31.00(467442)
バックグラウンド:3.430(627)　グループ 1 - イベント 1　Scan

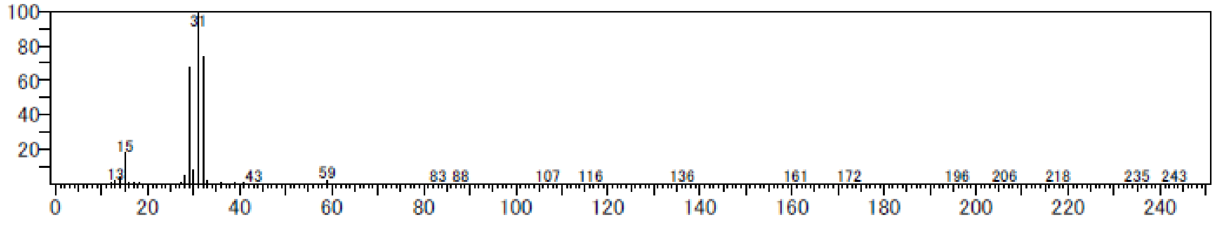

ヒット#:1　エントリ#:16　ライブラリ:NIST11.lib
SI:96　分子式:CH4O　CAS:67-56-1　分子量:32　保持指標:0
化合物名:Methyl Alcohol $$ Methanol $$ Carbinol $$ Methyl hydroxide $$ Methylol $$ Monohydroxymethane $$ Wood alcohol $$

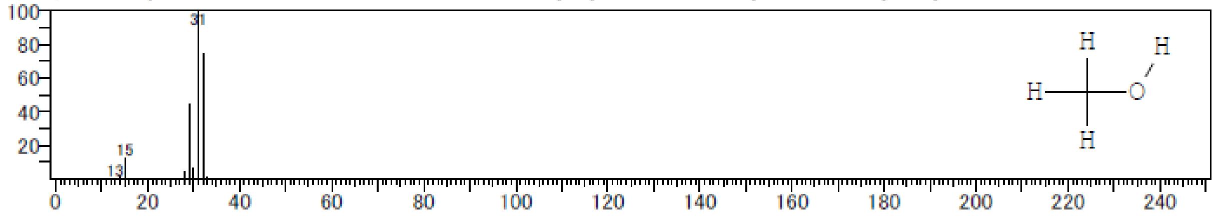

Compound 5

<< ターゲット >>
ライン#:5　保持時間:3.510(スキャン#:643)　ピーク数:139
スペクトル:Averaged 3.480-3.530(637-647)　ベースピーク:43.00(177436)
バックグラウンド:3.535(648)　グループ 1 - イベント 1　Scan

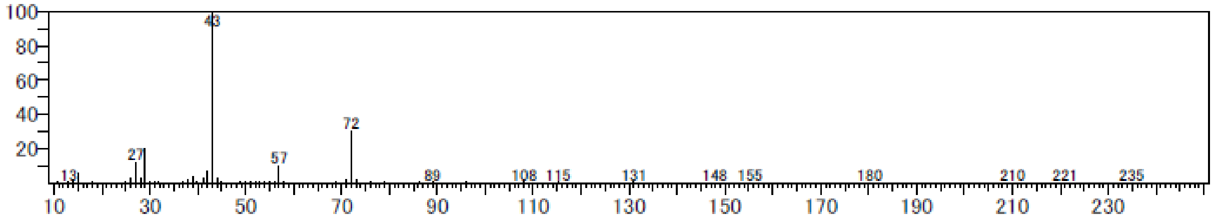

ヒット#:1　エントリ#:358　ライブラリ:NIST11s.lib
SI:96　分子式:C4H8O　CAS:78-93-3　分子量:72　保持指標:555
化合物名:2-Butanone $$ Butan-2-one $$ Butanone $$ Ethyl methyl ketone $$ Ketone, methyl ethyl $$ Methyl ethyl ketone $$ M

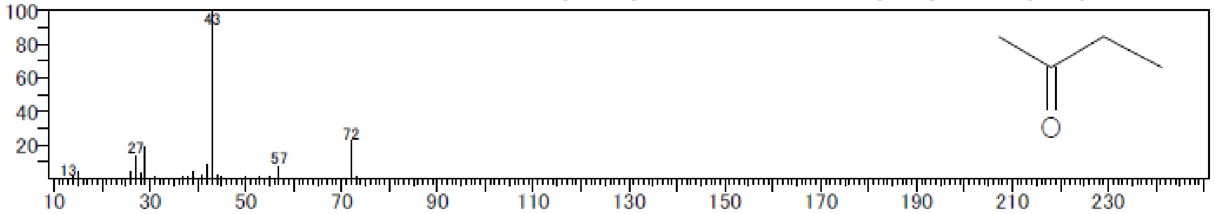

Compound 6

<< ターゲット >>
ライン#:6　保持時間:3.680(スキャン#:677)　ピーク数:126
スペクトル:Averaged 3.660-3.700(673-681)　ベースピーク:31.00(16947)
バックグラウンド:3.660(673)　グループ 1 - イベント 1　Scan

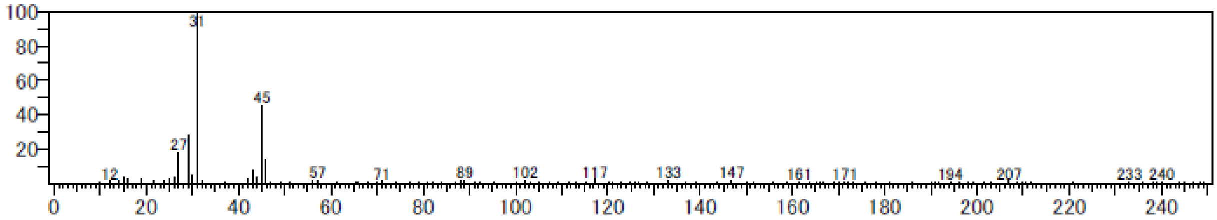

ヒット#:1　エントリ#:47　ライブラリ:NIST11s.lib
SI:88　分子式:C2H6O　CAS:64-17-5　分子量:46　保持指標:463
化合物名:Ethanol $$ Ethyl alcohol $$ Alcohol $$ Alcohol anhydrous $$ Algrain $$ Anhydrol $$ Denatured ethanol $$ Ethyl hydrat

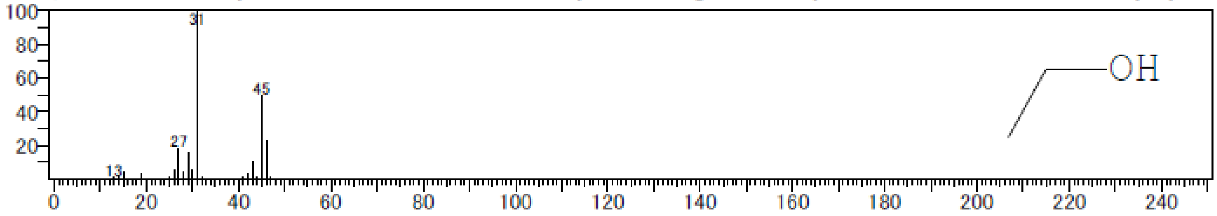

Compound 7

<< ターゲット >>
ライン#:7　保持時間:4.105(スキャン#:762)　ピーク数:153
スペクトル:Averaged 4.090-4.115(759-764)　ベースピーク:43.00(33069)
バックグラウンド:4.085(758)　グループ 1 - イベント 1　Scan

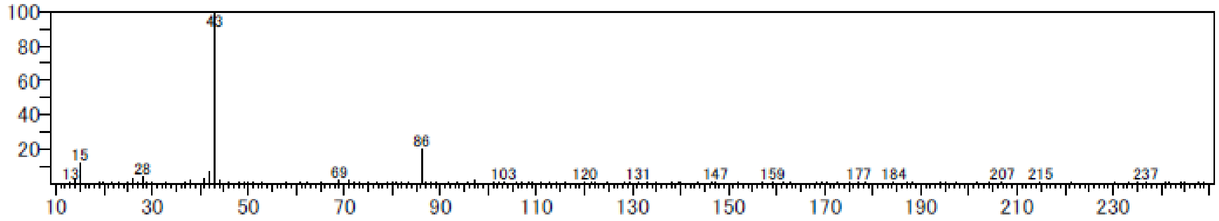

ヒット#:1　エントリ#:843　ライブラリ:NIST11s.lib
SI:90　分子式:C4H6O2　CAS:431-03-8　分子量:86　保持指標:691
化合物名:2,3-Butanedione $$ Biacetyl $$ Butane-2,3-dione $$ Butanedione $$ Diacetyl $$ Dimethyl diketone $$ Dimethyl glyoxa

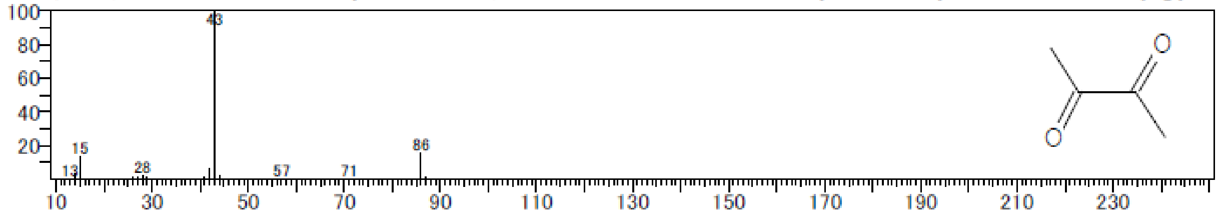

Compound 8

<< ターゲット >>
ライン#:8　保持時間:4.425(スキャン#:826)　ピーク数:239
スペクトル:Single 4.425(826)　ベースピーク:41.00(960394)
バックグラウンド:None　グループ 1 - イベント 1　Scan

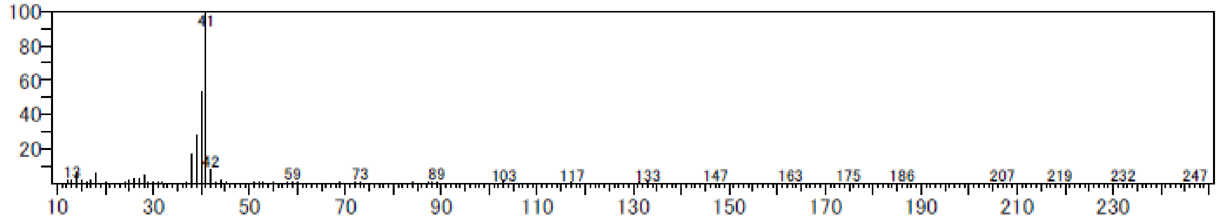

ヒット#:1　エントリ#:23　ライブラリ:NIST11s.lib
SI:94　分子式:C2H3N　CAS:75-05-8　分子量:41　保持指標:465
化合物名:Acetonitrile $$ Cyanomethane $$ Ethanenitrile $$ Ethyl nitrile $$ Methane, cyano- $$ Methanecarbonitrile $$ Methyl c

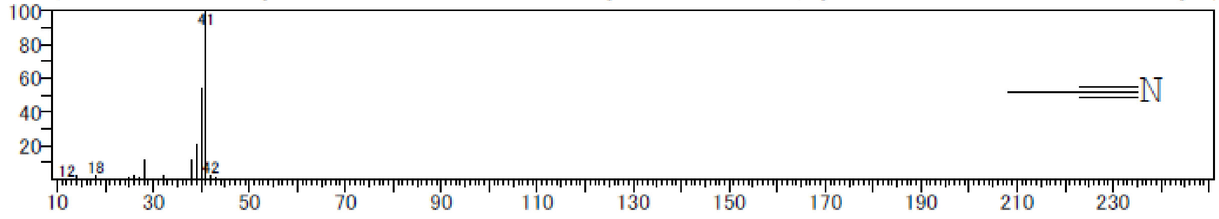

Compound 9

<< ターゲット >>
ライン#:9　保持時間:5.660(スキャン#:1073)　ピーク数:128
スペクトル:Averaged 5.645-5.680(1070-1077)　ベースピーク:41.00(19604)
バックグラウンド:5.645(1070)　グループ 1 - イベント 1　Scan

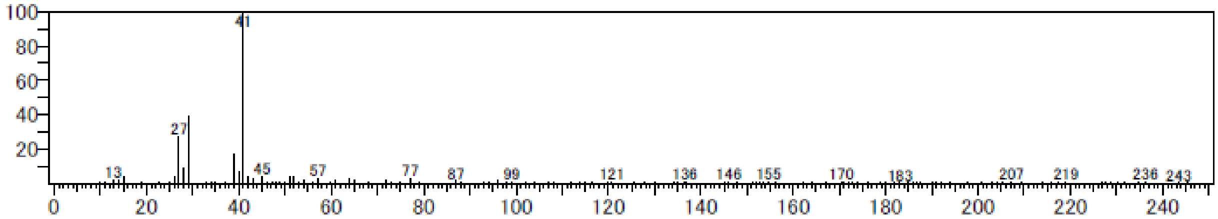

ヒット#:1　エントリ#:233　ライブラリ:NIST11.lib
SI:87　分子式:C4H7N　CAS:109-74-0　分子量:69　保持指標:664
化合物名:Butanenitrile $$ Butyronitrile $$ n-Butyronitrile $$ Butyrylonitrile $$ Propyl cyanide $$ 1-Cyanopropane $$ n-C3H7C

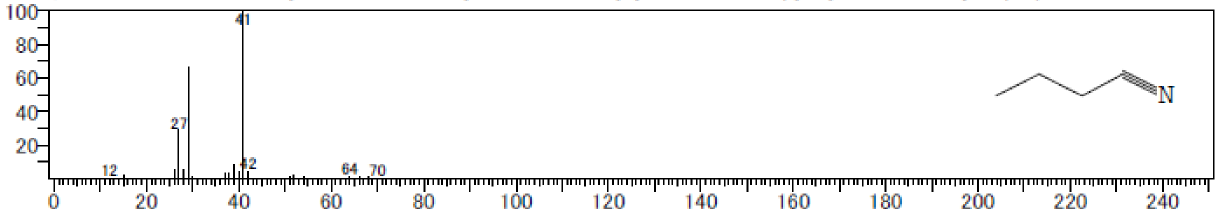

Compound 10

<< ターゲット >>
ライン#:10　保持時間:6.485(スキャン#:1238)　ピーク数:140
スペクトル:Averaged 6.470-6.505(1235-1242)　ベースピーク:41.00(18650)
バックグラウンド:6.460(1233)　グループ 1 - イベント 1　Scan

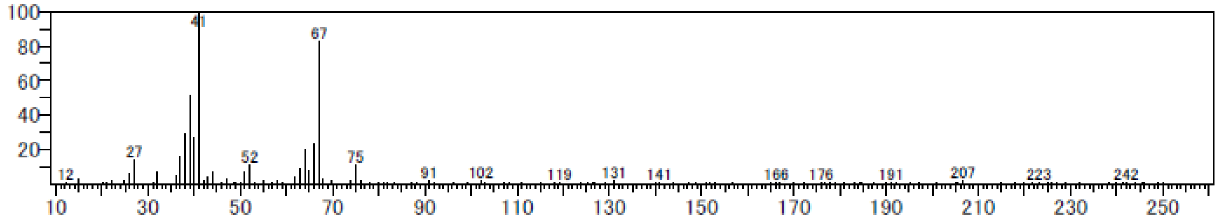

ヒット#:1　エントリ#:204　ライブラリ:NIST11s.lib
SI:89　分子式:C4H5N　CAS:4786-20-3　分子量:67　保持指標:672
化合物名:2-Butenenitrile $$ Crotononitrile $$ Crotonic nitrile $$ Crotonitrile $$ Crotonnitrile $$ 1-Cyanopropene $$ 1-Propenyl

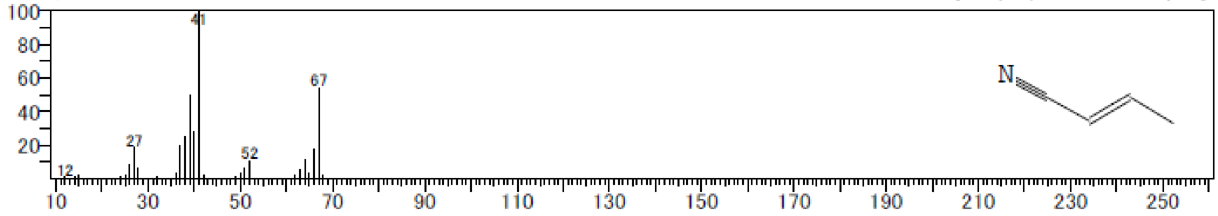

Compound 11

<< ターゲット >>
ライン#:11　保持時間:7.775(スキャン#:1496)　ピーク数:154
スペクトル:Averaged 7.760-7.795(1493-1500)　ベースピーク:59.00(23552)
バックグラウンド:7.755(1492)　グループ 1 - イベント 1　Scan

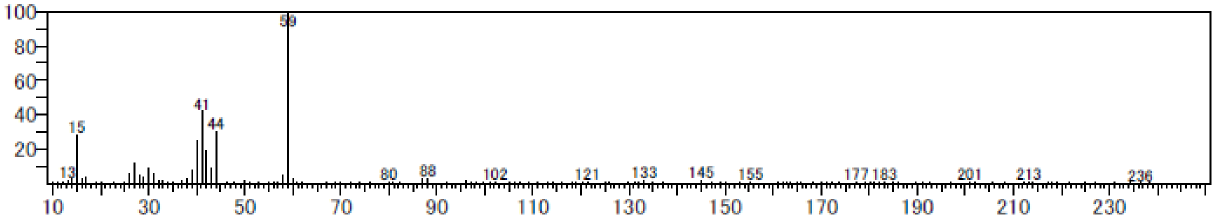

ヒット#:1　エントリ#:125　ライブラリ:NIST11s.lib
SI:87　分子式:C2H5NO　CAS:107-29-9　分子量:59　保持指標:575
化合物名:Acetaldoxime $$ (E)-CH3CH=NOH $$ Acetaldehyde oxime $$ Acetaldoxime,syn & anti $$ Aldoxime $$ Ethanal oxime $

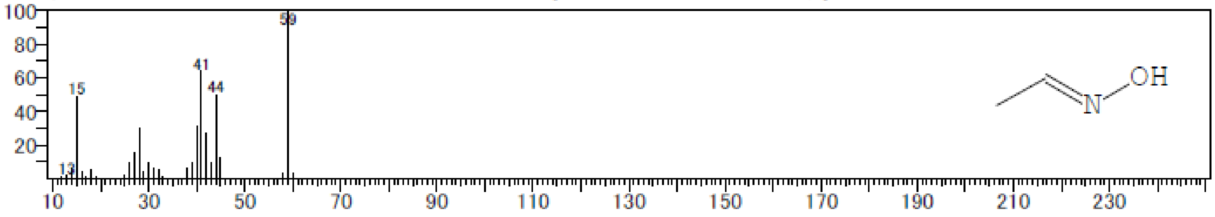

Compound 12

<< ターゲット >>
ライン#:12　保持時間:8.205(スキャン#:1582)　ピーク数:145
スペクトル:Averaged 8.190-8.215(1579-1584)　ベースピーク:82.05(52016)
バックグラウンド:8.220(1585)　グループ 1 - イベント 1　Scan

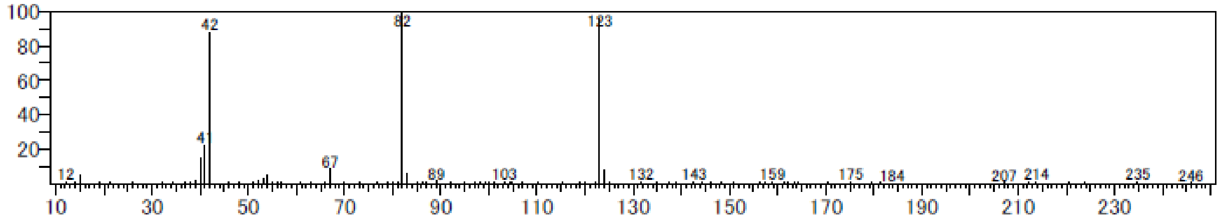

ヒット#:1　エントリ#:5713　ライブラリ:NIST11.lib
SI:95　分子式:C6H9N3　CAS:823-94-9　分子量:123　保持指標:1001
化合物名:1,3,5-Triazine, 2,4,6-trimethyl- $$ s-Triazine, 2,4,6-trimethyl- $$ Trimethyl-s-triazine $$ 2,4,6-Trimethyl-s-triazine $$

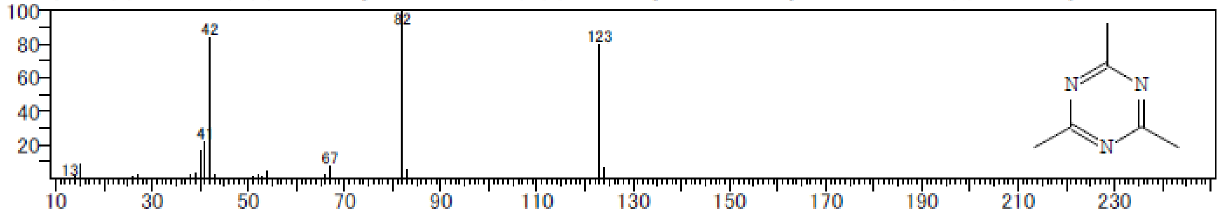

Compound 13

<< ターゲット >>
ライン#:13　保持時間:8.405(スキャン#:1622)　ピーク数:148
スペクトル:Averaged 8.390-8.425(1619-1626)　ベースピーク:45.00(52783)
バックグラウンド:8.430(1627)　グループ 1 - イベント 1　Scan

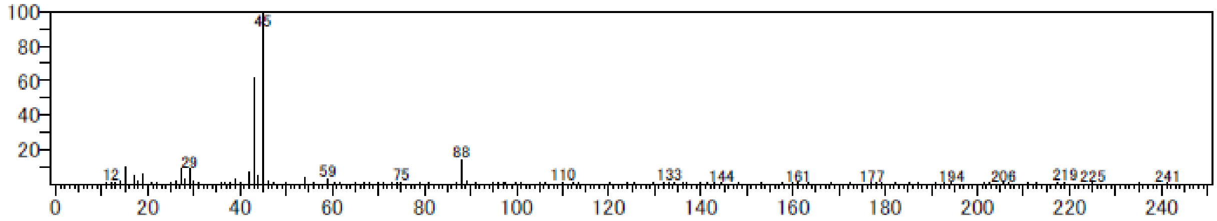

ヒット#:1　エントリ#:986　ライブラリ:NIST11.lib
SI:87　分子式:C4H8O2　CAS:513-86-0　分子量:88　保持指標:717
化合物名:Acetoin $$ 2-Butanone, 3-hydroxy- $$ .gamma.-Hydroxy-.beta.-oxobutane $$ Acetyl methyl carbinol $$ Dimethylketol

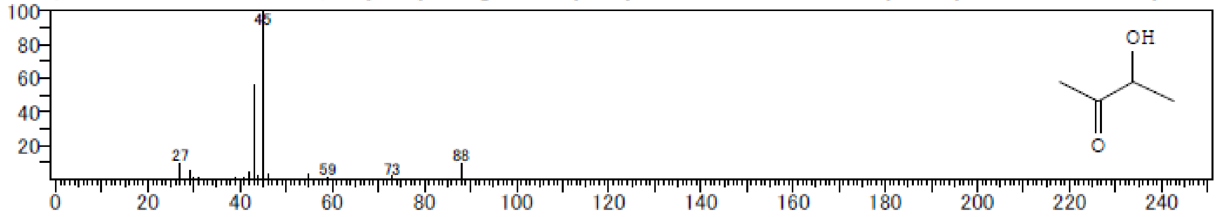

Compound 14

<< ターゲット >>
ライン#:14　保持時間:10.565(スキャン#:2054)　ピーク数:240
スペクトル:Single 10.565(2054)　ベースピーク:43.00(252826)
バックグラウンド:None　グループ 1 - イベント 1　Scan

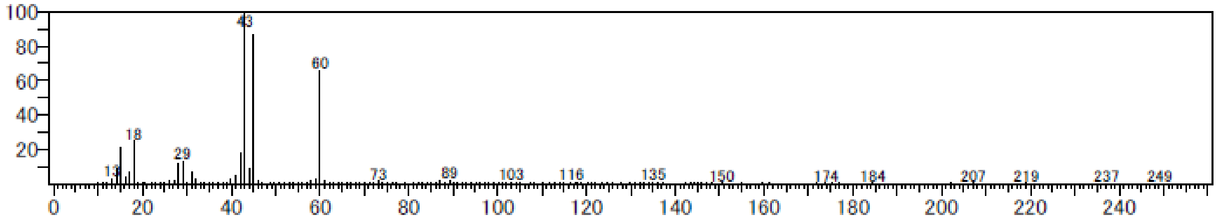

ヒット#:1　エントリ#:139　ライブラリ:NIST11s.lib
SI:92　分子式:C2H4O2　CAS:64-19-7　分子量:60　保持指標:576
化合物名:Acetic acid $$ Ethanoic acid $$ Ethylic acid $$ Glacial acetic acid $$ Methanecarboxylic acid $$ Vinegar acid $$ CH3C

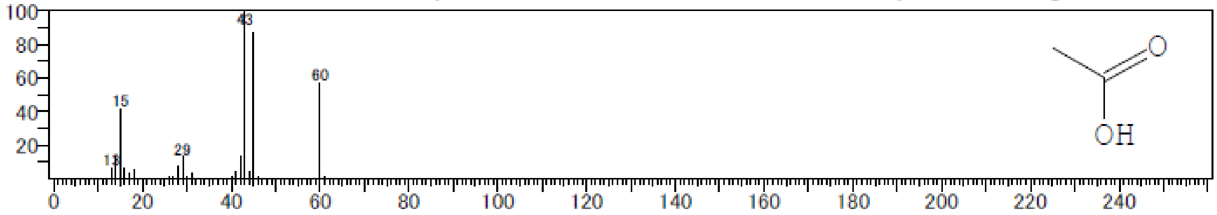

Compound 15

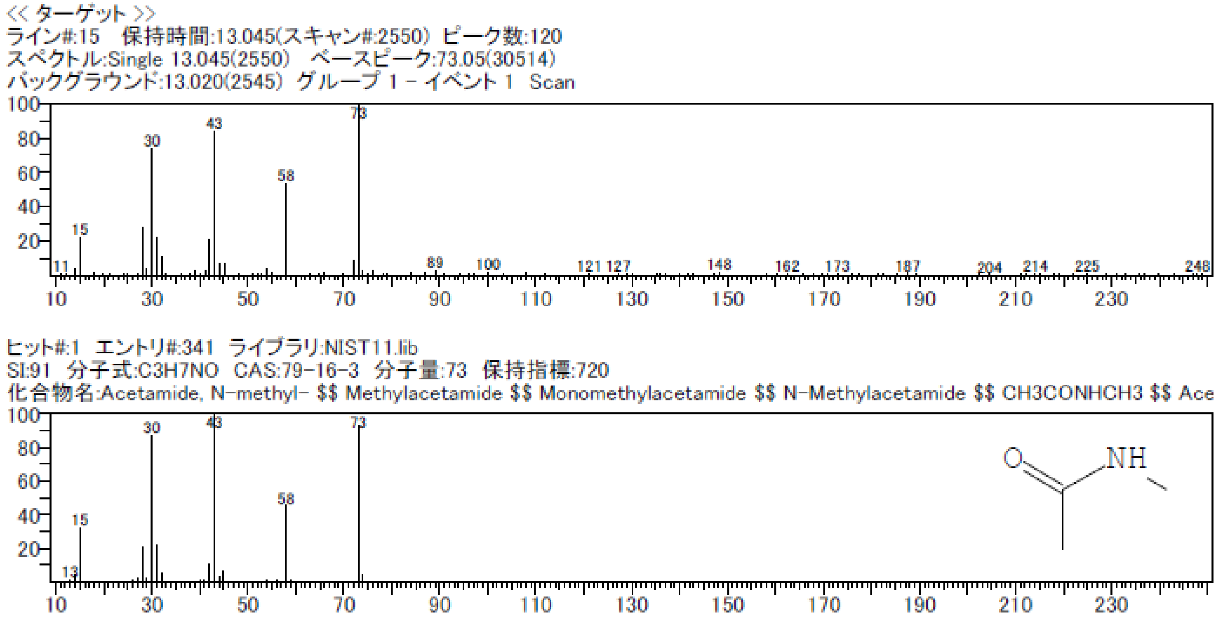

Compound 16

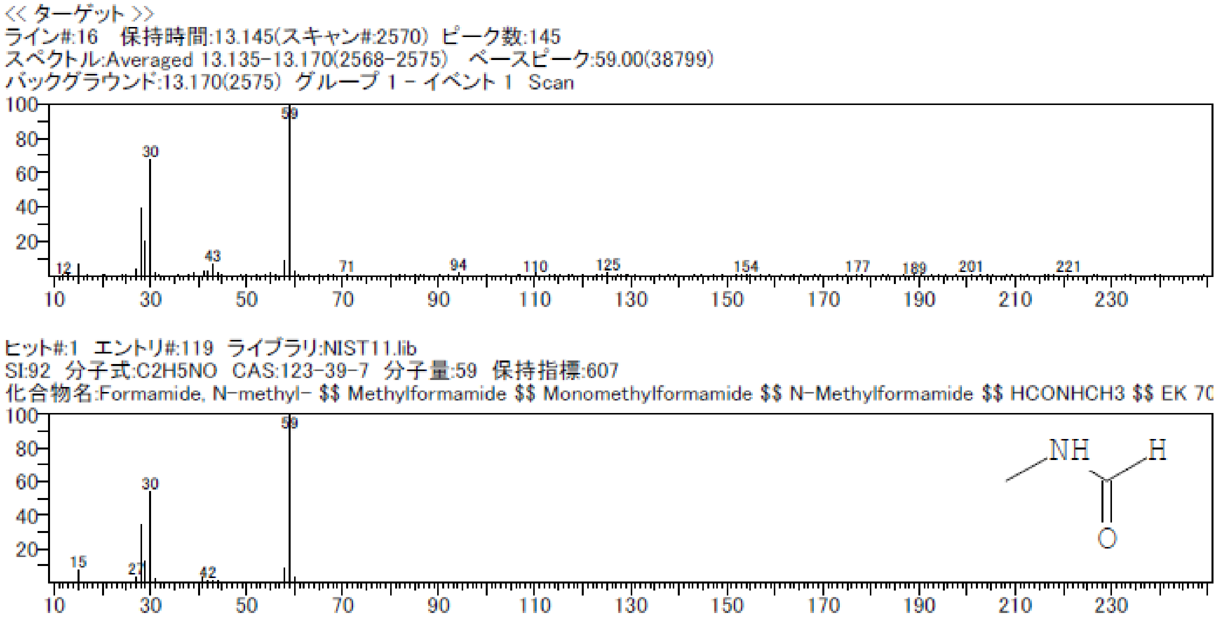

Compound 17

<< ターゲット >>
ライン#:17　保持時間:14.470(スキャン#:2835)　ピーク数:238
スペクトル:Single 14.470(2835)　ベースピーク:59.00(5172410)
バックグラウンド:None　グループ 1 - イベント 1　Scan

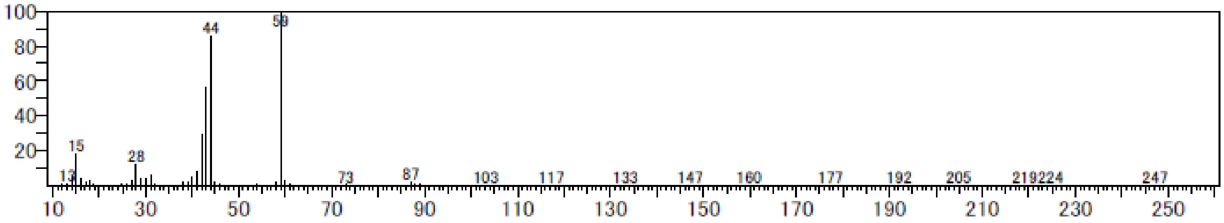

ヒット#:1　エントリ#:121　ライブラリ:NIST11.lib
SI:96　分子式:C2H5NO　CAS:60-35-5　分子量:59　保持指標:629
化合物名:Acetamide $$ Acetic acid amide $$ Ethanamide $$ Methanecarboxamide $$ CH3CONH2 $$ NCI-C02108 $$ Amid kysel

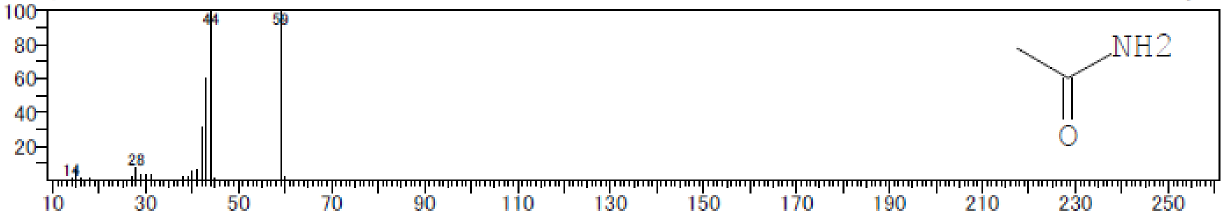

Compound 18

<< ターゲット >>
ライン#:18　保持時間:14.780(スキャン#:2897)　ピーク数:129
スペクトル:Single 14.780(2897)　ベースピーク:45.00(1205123)
バックグラウンド:14.735(2888)　グループ 1 - イベント 1　Scan

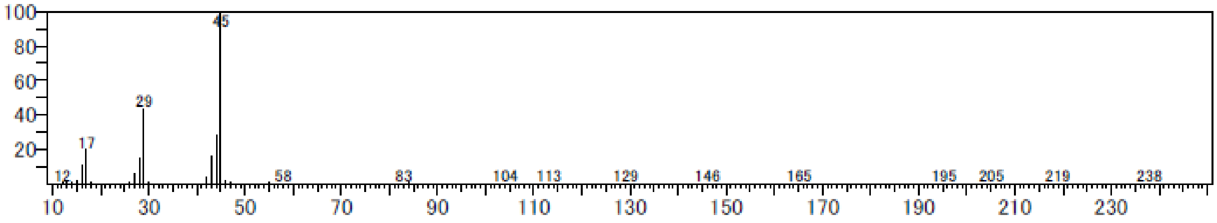

ヒット#:1　エントリ#:49　ライブラリ:NIST11.lib
SI:94　分子式:CH3NO　CAS:75-12-7　分子量:45　保持指標:0
化合物名:Formamide $$ Carbamaldehyde $$ Methanamide $$ HCONH2 $$ Amid kyseliny mravenci $$ Formimidic acid $$

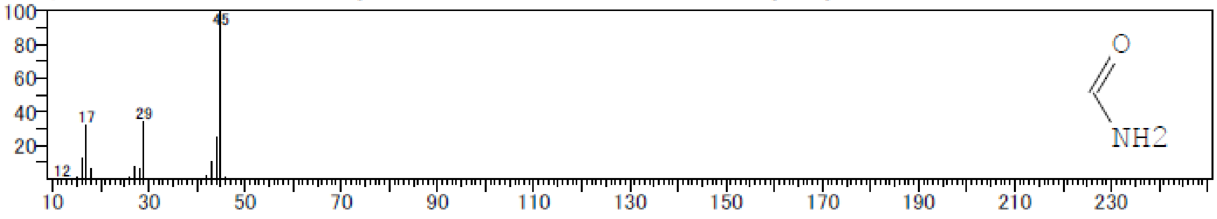

Compound 19

<< ターゲット >>
ライン#:19　保持時間:15.040(スキャン#:2949)　ピーク数:129
スペクトル:Single 15.040(2949)　ベースピーク:44.00(75657)
バックグラウンド:15.060(2953)　グループ 1 - イベント 1　Scan

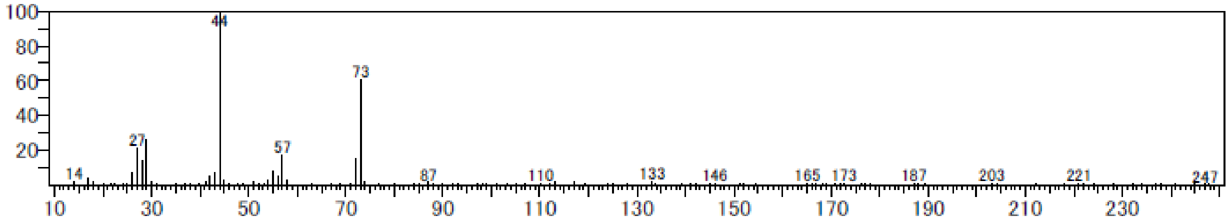

ヒット#:1　エントリ#:342　ライブラリ:NIST11.lib
SI:92　分子式:C3H7NO　CAS:79-05-0　分子量:73　保持指標:729
化合物名:Propanamide $$ Propionamide $$ Propionic acid amide $$ Propionic amide $$ Amid kyseliny propionove $$

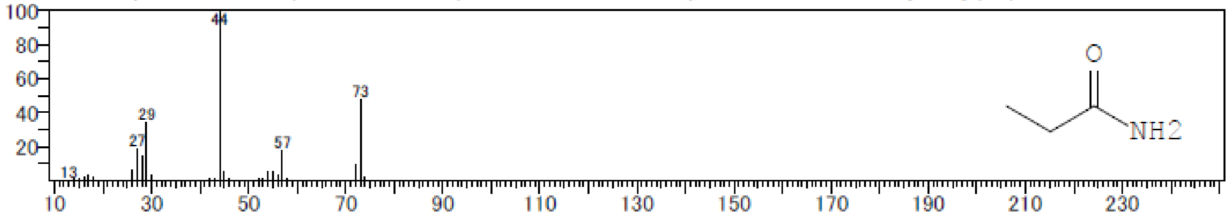

Compound 20

<< ターゲット >>
ライン#:20　保持時間:15.145(スキャン#:2970)　ピーク数:149
スペクトル:Averaged 15.120-15.160(2965-2973)　ベースピーク:43.00(77097)
バックグラウンド:15.115(2964)　グループ 1 - イベント 1　Scan

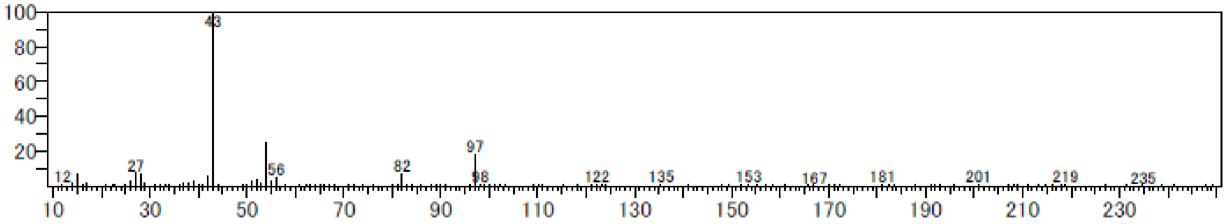

ヒット#:1　エントリ#:1492　ライブラリ:NIST11.lib
SI:86　分子式:C5H7NO　CAS:4468-47-7　分子量:97　保持指標:835
化合物名:2-Methyl-3-oxobutyronitrile $$ 2-Methyl-3-oxobutanenitrile # $$

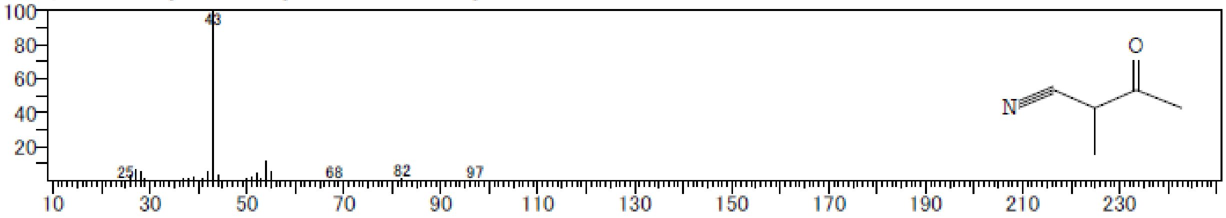

Compound 21

<< ターゲット >>
ライン#:21　保持時間:15.355(スキャン#:3012)　ピーク数:136
スペクトル:Averaged 15.325-15.370(3006-3015)　ベースピーク:43.00(67378)
バックグラウンド:15.400(3021)　グループ 1 - イベント 1　Scan

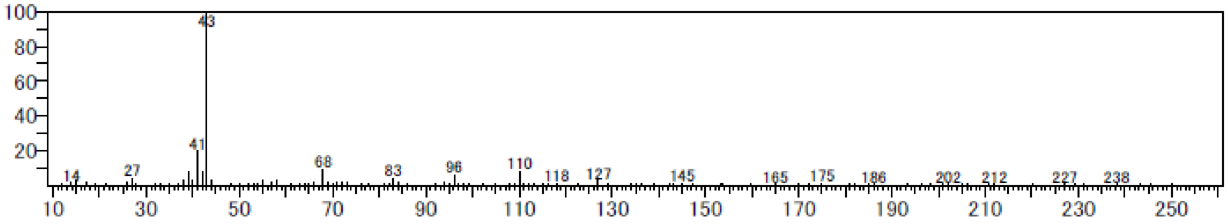

ヒット#:1　エントリ#:3293　ライブラリ:NIST11.lib
SI:90　分子式:C6H9NO　CAS:10412-98-3　分子量:111　保持指標:999
化合物名:5-Oxohexanenitrile $$ Hexanenitrile, 5-oxo- $$

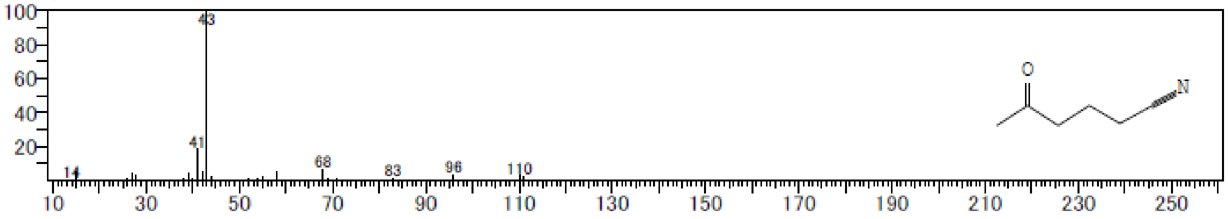

Compound 22

<< ターゲット >>
ライン#:22　保持時間:15.580(スキャン#:3057)　ピーク数:133
スペクトル:Averaged 15.575-15.590(3056-3059)　ベースピーク:45.00(61187)
バックグラウンド:15.600(3061)　グループ 1 - イベント 1　Scan

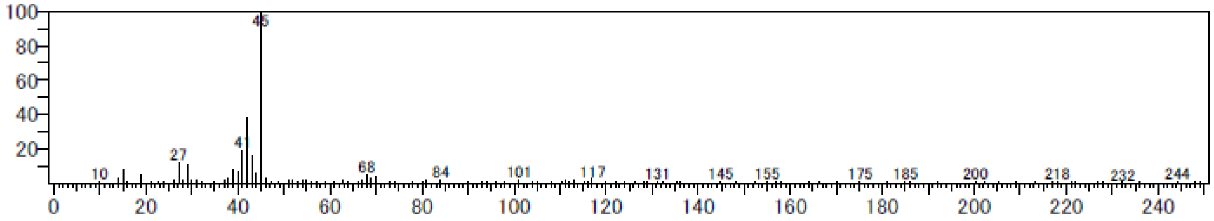

ヒット#:1　エントリ#:2106　ライブラリ:NIST11s.lib
SI:85　分子式:C6H14O　CAS:626-93-7　分子量:102　保持指標:780
化合物名:2-Hexanol $$ n-C4H9CH(OH)CH3 $$ n-Butylmethylcarbinol $$ Hexanol-(2) $$ sec-Hexyl alcohol $$ n-Hexan-2-ol $$

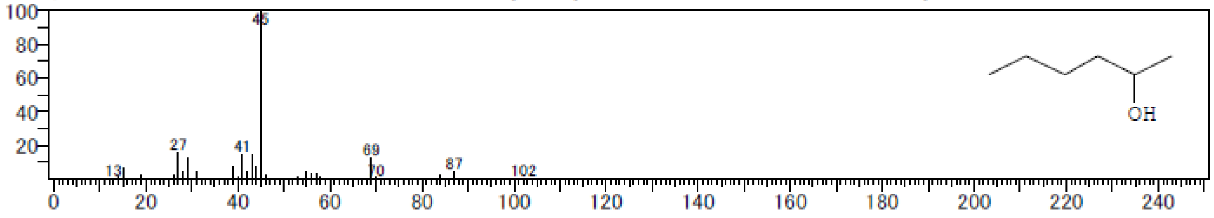

Compound 23

<< ターゲット >>
ライン#:23　保持時間:16.655(スキャン#:3272)　ピーク数:241
スペクトル:Single 16.655(3272)　ベースピーク:44.05(964500)
バックグラウンド:None　グループ 1 - イベント 1　Scan

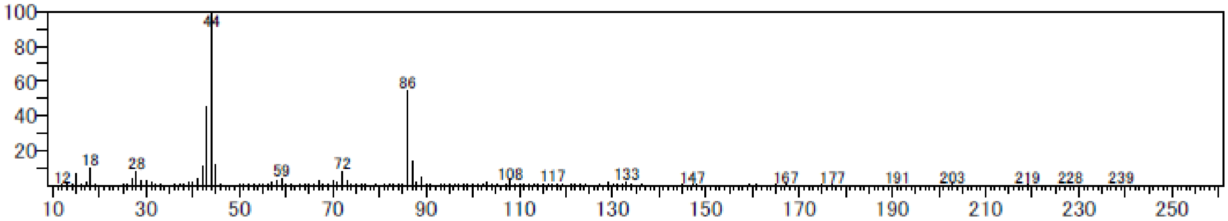

ヒット#:1　エントリ#:8139　ライブラリ:NIST11.lib
SI:89　分子式:C5H9NO3　CAS:1115-69-1　分子量:131　保持指標:1211
化合物名:DL-Alanine, N-acetyl- $$ 2-Acetylaminopropionic acid $$ N-Acetylalanine # $$

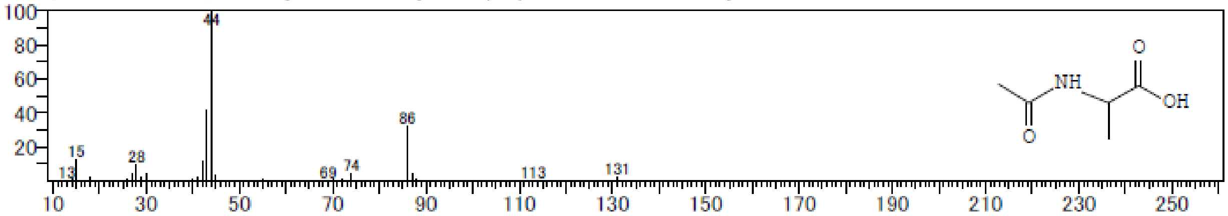

Compound 24

<< ターゲット >>
ライン#:24　保持時間:16.740(スキャン#:3289)　ピーク数:112
スペクトル:Averaged 16.715-16.755(3284-3292)　ベースピーク:31.00(33960)
バックグラウンド:16.770(3295)　グループ 1 - イベント 1　Scan

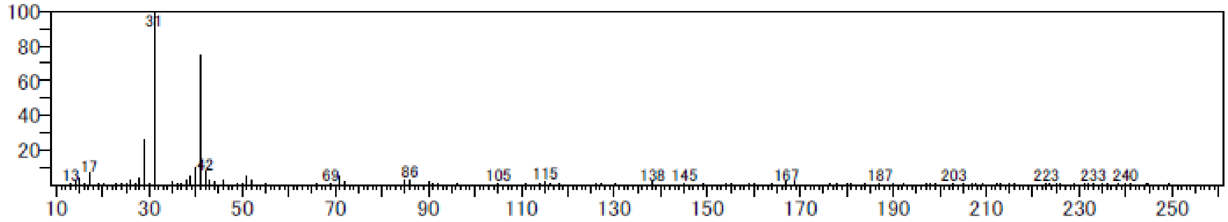

ヒット#:1　エントリ#:282　ライブラリ:NIST11.lib
SI:89　分子式:C3H5NO　CAS:109-78-4　分子量:71　保持指標:807
化合物名:Propanenitrile, 3-hydroxy- $$ Hydracrylonitrile $$ .beta.-Cyanoethanol $$ .beta.-Hydroxypropionitrile $$ Ethylene cyan

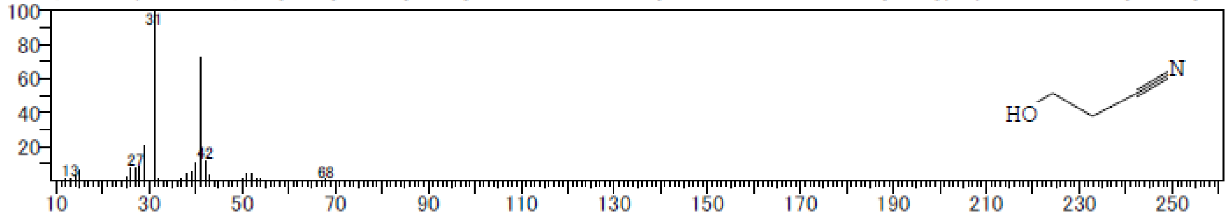

Compound 25

<< ターゲット >>
ライン#:25　保持時間:19.065(スキャン#:3754) ピーク数:132
スペクトル:Averaged 19.035-19.075(3748-3756)　ベースピーク:96.05(132047)
バックグラウンド:19.090(3759) グループ 1 - イベント 1　Scan

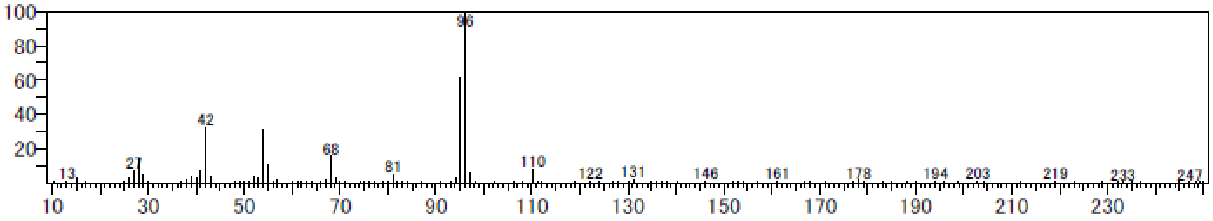

ヒット#:1　エントリ#:1383　ライブラリ:NIST11.lib
SI:92　分子式:C5H8N2　CAS:1739-84-0　分子量:96　保持指標:804
化合物名:1H-Imidazole, 1,2-dimethyl- $$ Imidazole, 1,2-dimethyl- $$ 1,2-Dimethylimidazole $$ 1,2-Dimethyl-1H-imidazole # $$ [illegible]

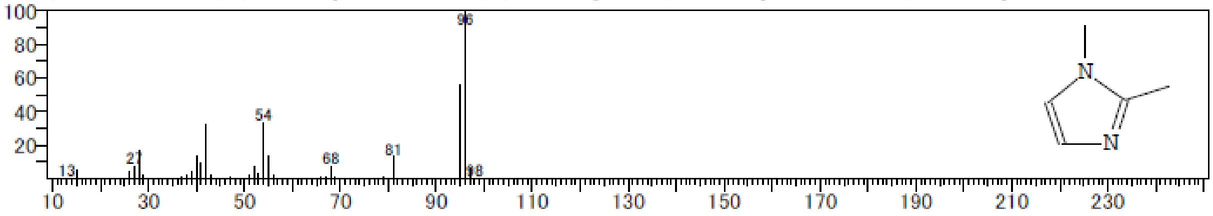

Compound 26

<< ターゲット >>
ライン#:26　保持時間:19.205(スキャン#:3782) ピーク数:113
スペクトル:Averaged 19.185-19.220(3778-3785)　ベースピーク:53.00(888980)
バックグラウンド:19.240(3789) グループ 1 - イベント 1　Scan

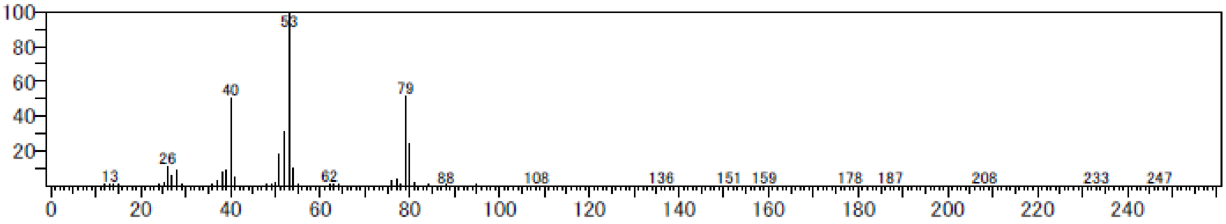

ヒット#:1　エントリ#:567　ライブラリ:NIST11s.lib
SI:97　分子式:C4H4N2　CAS:110-61-2　分子量:80　保持指標:909
化合物名:Butanedinitrile $$ Succinonitrile $$ s-Dicyanoethane $$ Deprelin $$ Dician $$ Dinile $$ Disuxyl $$ Ethane, 1,2-dicyano

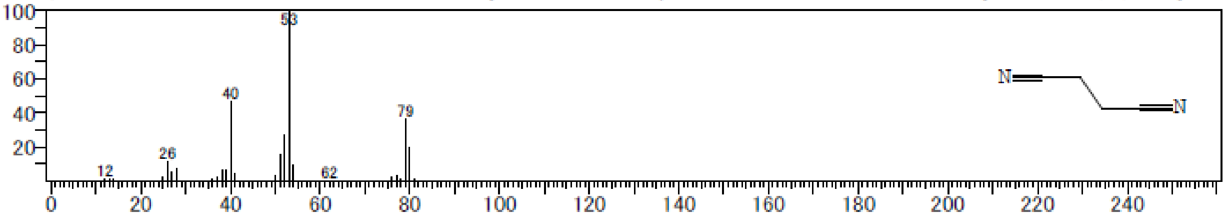

Compound 27

<< ターゲット >>
ライン#:27 保持時間:19.440(スキャン#:3829) ピーク数:144
スペクトル:Averaged 19.430-19.450(3827-3831) ベースピーク:42.00(96680)
バックグラウンド:19.420(3825) グループ 1 - イベント 1 Scan

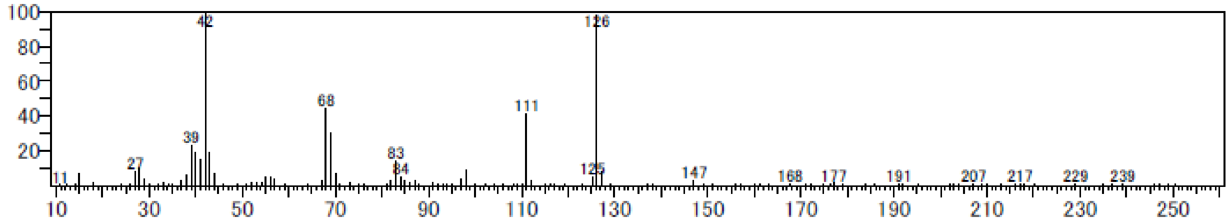

ヒット#:1 エントリ#:4711 ライブラリ:NIST11s.lib
SI:81 分子式:C5H6N2O2 CAS:626-48-2 分子量:126 保持指標:1271
化合物名:6-Methyluracil $$ 2,4(1H,3H)-Pyrimidinedione, 6-methyl- $$ 2(1H)-Pyrimidinone, 4-hydroxy-6-methyl- $$ Pseudothym

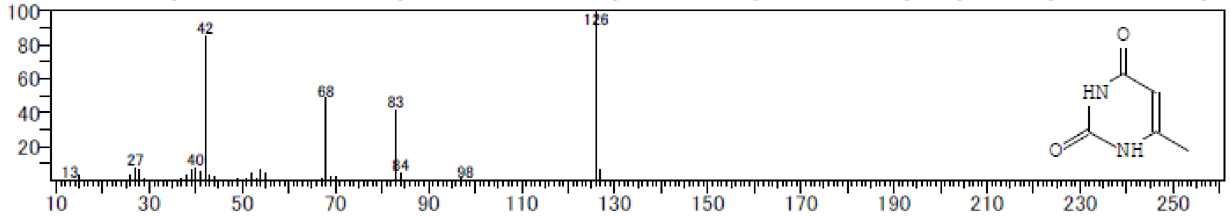

Compound 28

<< ターゲット >>
ライン#:28 保持時間:19.675(スキャン#:3876) ピーク数:129
スペクトル:Averaged 19.670-19.705(3875-3882) ベースピーク:110.10(385296)
バックグラウンド:19.705(3882) グループ 1 - イベント 1 Scan

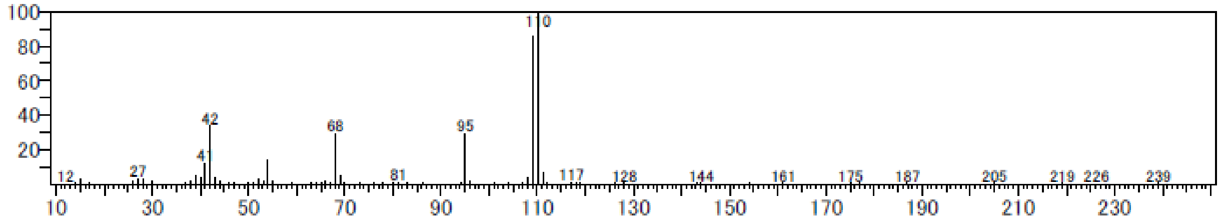

ヒット#:1 エントリ#:3090 ライブラリ:NIST11.lib
SI:90 分子式:C6H10N2 CAS:5519-42-6 分子量:110 保持指標:1100
化合物名:3,4,5-Trimethylpyrazole $$ 1H-Pyrazole, 3,4,5-trimethyl- $$ 3,4,5-Trimethyl-1H-pyrazole # $$

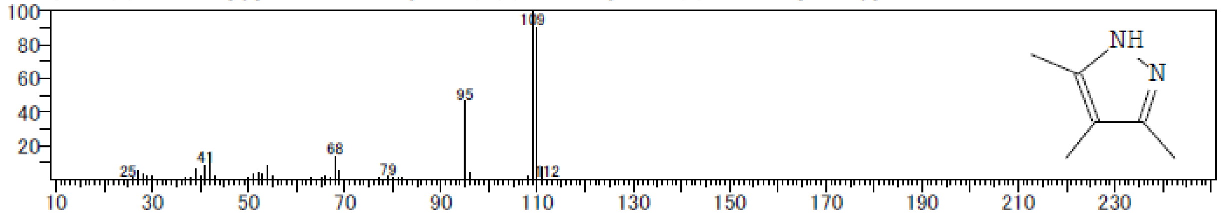

Compound 29

<< ターゲット >>
ライン#:29　保持時間:20.345(スキャン#:4010)　ピーク数:132
スペクトル:Averaged 20.325-20.360(4006-4013)　ベースピーク:45.00(135523)
バックグラウンド:20.365(4014)　グループ 1 - イベント 1　Scan

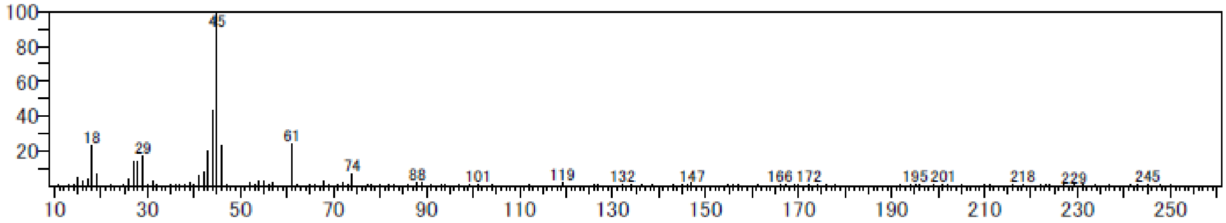

ヒット#:1　エントリ#:1066　ライブラリ:NIST11.lib
SI:92　分子式:C3H7NO2　CAS:2043-43-8　分子量:89　保持指標:891
化合物名:Propanamide, 2-hydroxy- $$ Lactamide $$ .alpha.-Hydroxypropionamide $$ Lactic acid amide $$ Lactic amide $$ 2-Hy

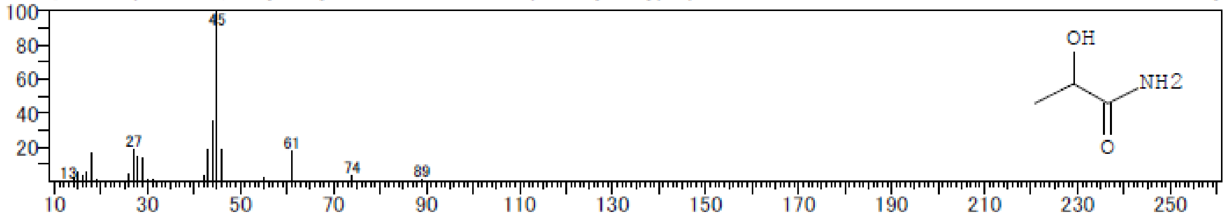

Compound 30

<< ターゲット >>
ライン#:30　保持時間:21.335(スキャン#:4208)　ピーク数:140
スペクトル:Averaged 21.320-21.350(4205-4211)　ベースピーク:42.00(194052)
バックグラウンド:21.310(4203)　グループ 1 - イベント 1　Scan

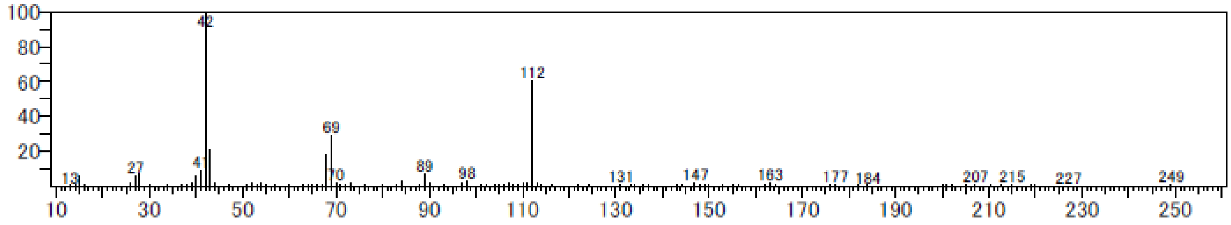

ヒット#:1　エントリ#:2853　ライブラリ:NIST11s.lib
SI:79　分子式:C4H4N2O2　CAS:66-22-8　分子量:112　保持指標:1109
化合物名:Uracil $$ 2,4(1H,3H)-Pyrimidinedione $$ Pirod $$ Pyrod $$ RU 12709 $$ Ura $$ 2,4-Dihydroxypyrimidine $$ 2,4-Dioxop

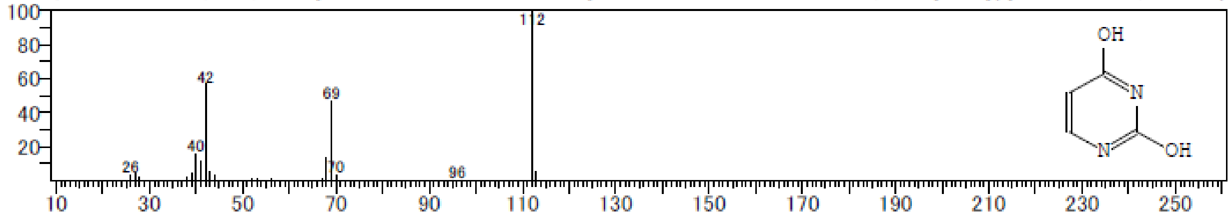

Compound 31

<< ターゲット >>
ライン#:31　保持時間:22.285(スキャン#:4398) ピーク数:115
スペクトル:Averaged 22.255-22.305(4392-4402)　ベースピーク:44.00(90800)
バックグラウンド:22.255(4392) グループ 1 - イベント 1　Scan

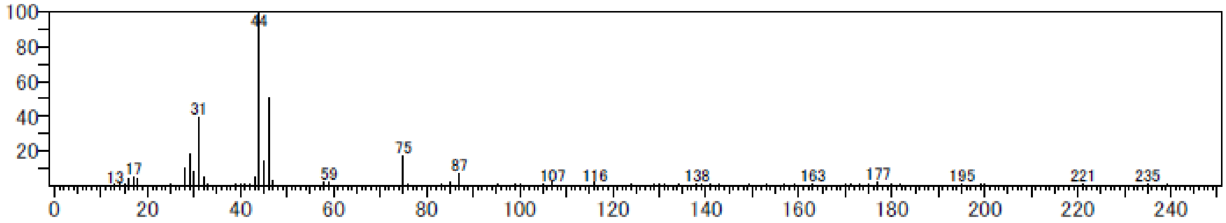

ヒット#:1　エントリ#:476　ライブラリ:NIST11s.lib
SI:86　分子式:C2H5NO2　CAS:598-55-0　分子量:75　保持指標:630
化合物名:Carbamic acid, methyl ester $$ Methyl carbamate $$ Methylurethane $$ Urethylane $$ Methylurethan $$ NCI-C55594 :

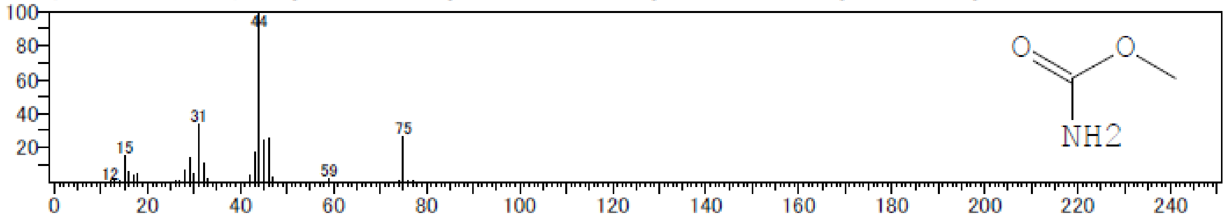

Supplement: Supplementary file 1 — Supplementary Information [file 41598_2017_18483_MOESM1_ESM.pdf]
